# Montmorillonite Interfacial Chemistry Regulation on Homogeneous Zn Deposition: A Microenvironment‐Controlled Additive Strategy for Sustainable Zinc Metal Anodes

**DOI:** 10.1002/smsc.202500377

**Published:** 2025-10-16

**Authors:** Hailong Xuan, Xiaolong Cheng, Yu Yao, Yihong Gao, Pengcheng Shi, Fangzhi Huang, Yu Jiang, Yan Yu

**Affiliations:** ^1^ School of Materials Science and Engineering Anhui University Hefei 230601 China; ^2^ Hefei National Research Center for Physical Sciences at the Microscale Department of Materials Science and Engineering University of Science and Technology of China Hefei Anhui 230026 China; ^3^ School of Chemistry and Chemical Engineering Anhui University Hefei 230601 China

**Keywords:** aqueous zinc metal batteries, dendrite‐free deposition, electrolyte additives, phytic acid‐functionalized montmorillonite, zinc anodes

## Abstract

Aqueous zinc (Zn) metal batteries (AZBs) have emerged as highly promising candidates for large‐scale energy storage systems because of their inherent safety and cost‐effectiveness. However, their practical implementation remains constrained by parasitic side reactions and uncontrolled dendrite growth at the metallic Zn anode. Herein, a microenvironment‐controlled additive strategy is proposed via employing phytic acid‐functionalized montmorillonite (MPA) nanosheets as electrolyte additives for highly durable AZBs. The MPA nanosheets spontaneously assemble onto the surface of the Zn anode through interfacial self‐adsorption, effectively suppressing parasitic reactions. Moreover, the regulation of interfacial chemistry enhances the zincophilic characteristic, enabling precise modulation of Zn^2+^ flux distribution and directing homogeneous Zn electrodeposition through spatially controlled ion coordination. As a result, the Zn||Zn symmetric cell with the MPA additives achieves a stable cycle for over 2800 h at 2 mA cm^−2^. The assembled Zn||VO_2_ full cell within the modified electrolyte maintains exceptional cycling stability of 89.5% after 1000 cycles. This work presents a facile and efficient microenvironment‐regulated additive strategy for homogeneous Zn deposition, aimed at achieving highly reversible AZBs.

## Graphical abstract

Highly durable Zn metal batteries are achieved by employing phytic acid‐functionalized montmorillonite (MPA) nanosheets as electrolyte additives. The MPA nanosheets spontaneously self‐assemble onto the Zn surface, forming a protective layer that enables multilevel regulation of the Zn^2+^ deposition process. This layer suppresses parasitic reactions and enables precise modulation of Zn^2+^ flux distribution, leading to dendrite‐free Zn deposition.

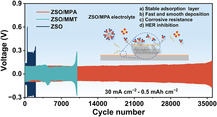

## 1 Introduction

Rechargeable aqueous Zn metal batteries (AZBs) have been regarded as one of the most promising next‐generation energy storage systems because of their inherent properties, including low cost, considerable theoretical capacity (5855 mAh cm^−3^, 820 mAh g^−1^), and high safety.^[^ ^1^, ^2^ ^]^ However, the uncontrollable growth of Zn dendrites and the continuous hydrogen evolution reaction (HER) at the Zn anode interface during Zn deposition severely limit the practical application of AZBs.^[^ ^3^, ^4^ ^]^ Therefore, regulating the Zn deposition behavior is of great significance in enhancing the utilization rate and extending the cycle life of the Zn anode.^[^ ^5^ ^]^

The perplexing challenges are fundamentally correlated with the coordination environment of Zn‐ions, solvent molecules, and anions, particularly the solvation structure adjacent to the electrolyte‐anode interface.^[^ ^6^, ^7^ ^]^ During charging, these solvated species undergo desolvation and electron transfer at the interface, resulting in heterogeneous deposition. Resolving these issues necessitates dual regulation of Zn^2+^ solvation shell and interfacial optimization to achieve uniform Zn plating/stripping with accelerated reaction kinetics.^[^ ^8^, ^9^ ^]^ Thus, developing facile yet efficient strategies to address these interfacial challenges becomes imperative, ensuring long‐term stability of Zn metal anodes and enabling the practical development of AZBs in large‐scale energy storage applications.^[^ ^10^ ^]^

To address these issues associated with the Zn^2+^ desolvation process and solid–liquid interfacial dynamics, researchers have developed multiple approaches, including Zn metal interface engineering,^[^ ^11^ ^]^ separator modification,^[^ ^12^ ^]^ and electrolyte additive regulation.^[^ ^13^ ^]^ Among these, the regulation of electrolyte additives is particularly promising and practical due to its remarkable optimization effect, scalability for industrial processes, and cost‐effectiveness.^[^ ^14^, ^15^ ^]^ Notably, most electrolyte additives predominantly exist in aqueous electrolytes as free molecular or ionic species, primarily functioning through the regulation solvation sheath of Zn‐ions or surface modulation of Zn metal anodes (ZMAs) via molecular adsorption or sacrificial decomposition.^[^ ^16^ ^]^ However, the inherent vulnerability of these additives to progressive depletion during extended cycling inevitably diminishes their electrochemical optimization effects in the AZBs. This critical limitation necessitates the urgent development of next‐generation additives capable of concurrently accelerating the Zn^2+^ desolvation process and establishing self‐sustaining interfacial layers to ensure perpetual cycling stability.^[^ ^17^ ^]^

Building on these insights, a novel class of insoluble electrolyte additives has emerged as a strategic solution to circumvent progressive depletion and maintain interfacial passivation throughout extended cycling operations, including C_3_N_4_ nanosheets,^[^ ^18^ ^]^ zirconium phosphate nanosheets,^[^ ^19^ ^]^ carbon quantum dots,^[^ ^20^ ^]^ black phosphorus,^[^ ^21^ ^]^ and others. However, these materials are still constrained by complex synthesis protocols, which impede their scalable production for industrial battery manufacturing. Montmorillonite (MMT) has garnered widespread interest in the energy storage field due to its low cost, abundant resources, and unique ionic conductive properties, which are derived from its unique 2D crystal structure. Additionally, MMT features an intrinsic lamellar architecture with abundant surface functional groups that can be strategically tailored through surface modification strategies to achieve targeted functionalities.^[^ ^22^, ^23^, ^24^ ^]^

Herein, we propose a microenvironment‐controlled additive strategy by incorporating phytic acid (PA)‐functionalized montmorillonite (MPA) nanosheets as electrolyte additives into the 2 M ZnSO_4_ aqueous electrolyte to enable highly durable AZBs. The surface‐optimized MMT exhibits amplified Zn^2+^ chemisorption affinity and enhanced chelation capabilities, and it also demonstrates significant advantages in terms of both cost and preparation process (Table S1, Supporting Information). When employed as an electrolyte additive, the elaborated MPA nanosheets spontaneously self‐assemble onto the Zn surface through interfacial chemisorption, forming a protective layer that enables multilevel regulation of the Zn^2+^ deposition process. The obtained MPA‐based interphase layer effectively suppresses electrolyte‐induced corrosion and dendritic growth. Meanwhile, the optimized microenvironment enhances the zincophilicity of the interfacial layer, enabling the homogenization and distribution of Zn^2+^, leading to dendrite‐free electrodeposition. Benefiting from these merits, the Zn||Zn symmetric cell with the MPA additives exhibits a long cycling performance of over 2800 h at 2 mA cm^−2^. Moreover, the Zn asymmetrical cell with MPA additives achieves an average Coulombic efficiency (CE) of 99.9% after 2000 cycles at 10 mA cm^−2^. Additionally, the Zn||VO_2_ full cell operates stably for 1000 cycles and maintains an excellent capacity retention of 89.5%.

## 2 Results and Discussion

MMT is a natural silicate material characterized by a large number of hydroxyl (OH^−^) groups and a layered structure composed of aluminum octahedra sandwiched between two layers of silica tetrahedra (Figure S1, Supporting Information).^[^ ^25^ ^]^ Consequently, commercially available MMT was exfoliated into ultrathin nanosheets to significantly enhance its dispersibility in the aqueous electrolyte. X‐ray diffraction (XRD) analysis confirms a shift in the MMT (001) diffraction peak toward lower angles following exfoliation treatment, suggesting an increase in crystal spacing (Figure S2, Supporting Information).^[^ ^26^ ^]^ Characterization of the MMT using scanning/transmission electron microscopy (SEM) confirmed the successful exfoliation of MMT into nanosheets. The results from atomic force microscopy (AFM) showed that after undergoing exfoliation treatment, the thickness of the exfoliated MMT is ≈4 nm, suggesting that the MMT has primarily exfoliated into few‐layer nanosheets (2‐3 layers) (Figure S3, Supporting Information). The exfoliated MMT nanosheets were then modified with PA to obtain MPA nanosheets. Based on inductively coupled plasma analysis, the loading amount of PA was determined to be ≈2.5% (Figure S4, Supporting Information). The various PA contents of the MPA additives were successfully prepared. As shown in Figure S5, Supporting Information, the electrolyte with MPA‐1.0% additives delivers the longest cycle life compared to others, suggesting that optimizing the PA content of the MPA additives is an effective method to improve the electrochemical performance of the Zn anode.

**Figure 1:**
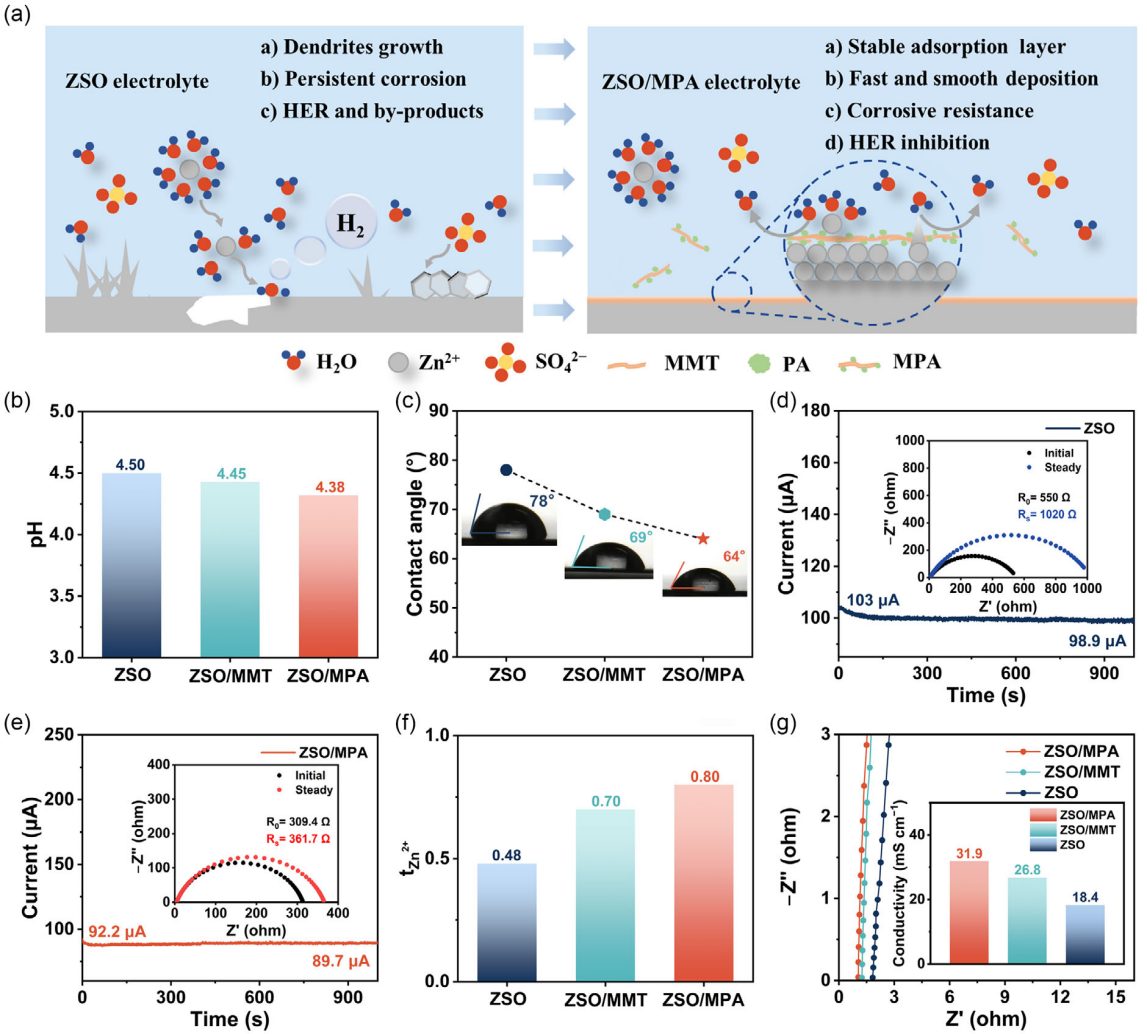
a) A schematic illustration of Zn deposition in ZSO and ZSO/MPA electrolytes. b) The pH of different electrolytes at room temperature. c) Contact angles on Zn anodes with different electrolytes. CA curves of Zn||Zn symmetric cells in d) ZSO and e) ZSO/MPA electrolytes. The inset shows the EIS profiles before and after polarization. f) Comparison of Zn^2+^ transference numbers. g) Ionic conductivity of different electrolytes.

The modified electrolyte was prepared via dispersing MPA nanosheets into a 2 mol L^−1^ ZnSO_4_ aqueous solution (ZSO electrolyte), named ZSO/MPA electrolytes. In comparison, the electrolyte with MMT nanosheets as an additive (ZSO/MMT) and the bare ZSO electrolyte were set as control groups. The optimal concentration of MPA additives was determined to be 0.15 g L^−1^ through a systematic evaluation of ionic conductivity, electrochemical impedance spectroscopy (EIS), and linear sweep voltammetry (LSV) (Figure S6, Supporting Information). Utilizing their unique crystal structure and clarified surface microenvironment, MPA nanosheets were employed as electrolyte additives for the AZBs, achieving multifunctional effects (**Figure** **1**a). In the ZSO/MPA electrolyte, the MPA nanosheets are adsorbed onto the Zn anode because of the interaction between the modified phosphoric acid groups and Zn metal, forming a stable artificial SEI layer (Figure S7, Supporting Information).^[^ ^27^ ^]^ The MPA‐based protection layer effectively blocks direct contact between the aqueous electrolyte and Zn metal, suppressing ongoing side reactions and the formation of harmful byproducts. The unique structure of MMT nanosheets can provide orderly and rapid channels for Zn^2+^ migration, thereby accelerating the Zn^2+^ transport kinetics.^[^ ^28^ ^]^ Furthermore, MPA nanosheets with an elaborated surface microenvironment exhibit enhanced zincophilicity, effectively homogenizing the Zn^2+^ flux. This leads to uniform stripping and deposition of Zn^2+^ on the Zn anode, thereby suppressing the formation of dendrites.

To verify the aforementioned design philosophy, comprehensive characterizations of the physicochemical properties of various electrolytes were conducted. Digital photographs of the electrolyte confirmed that the MMT and MPA nanosheets were stable and uniformly dispersed within the aqueous ZnSO_4_ solution, as evidenced by the Tyndall effect (Figure S8, Supporting Information). An appropriate chemical environment is crucial for ensuring the longevity of Zn batteries, in which an over‐acidic environment can significantly accelerate the corrosion process of Zn anode and exacerbate the occurrence of HERs.^[^ ^29^ ^]^ Figure 1b indicates that no significant change in pH value was observed after introducing trace additives into the electrolytes. Subsequently, the impact of the additives on the wettability of electrolytes toward Zn anode was investigated. As depicted in Figure 1c, upon the addition of MPA, the contact angle of the electrolyte decreased from 78° to 64°, indicating optimized surface wettability for Zn metal.^[^ ^30^, ^31^ ^]^ The enhanced wettability of the modified electrolyte is due to the strong chelating ability of the PO_4_^3−^ group from PA.^[^ ^32^ ^]^

**Figure 2:**
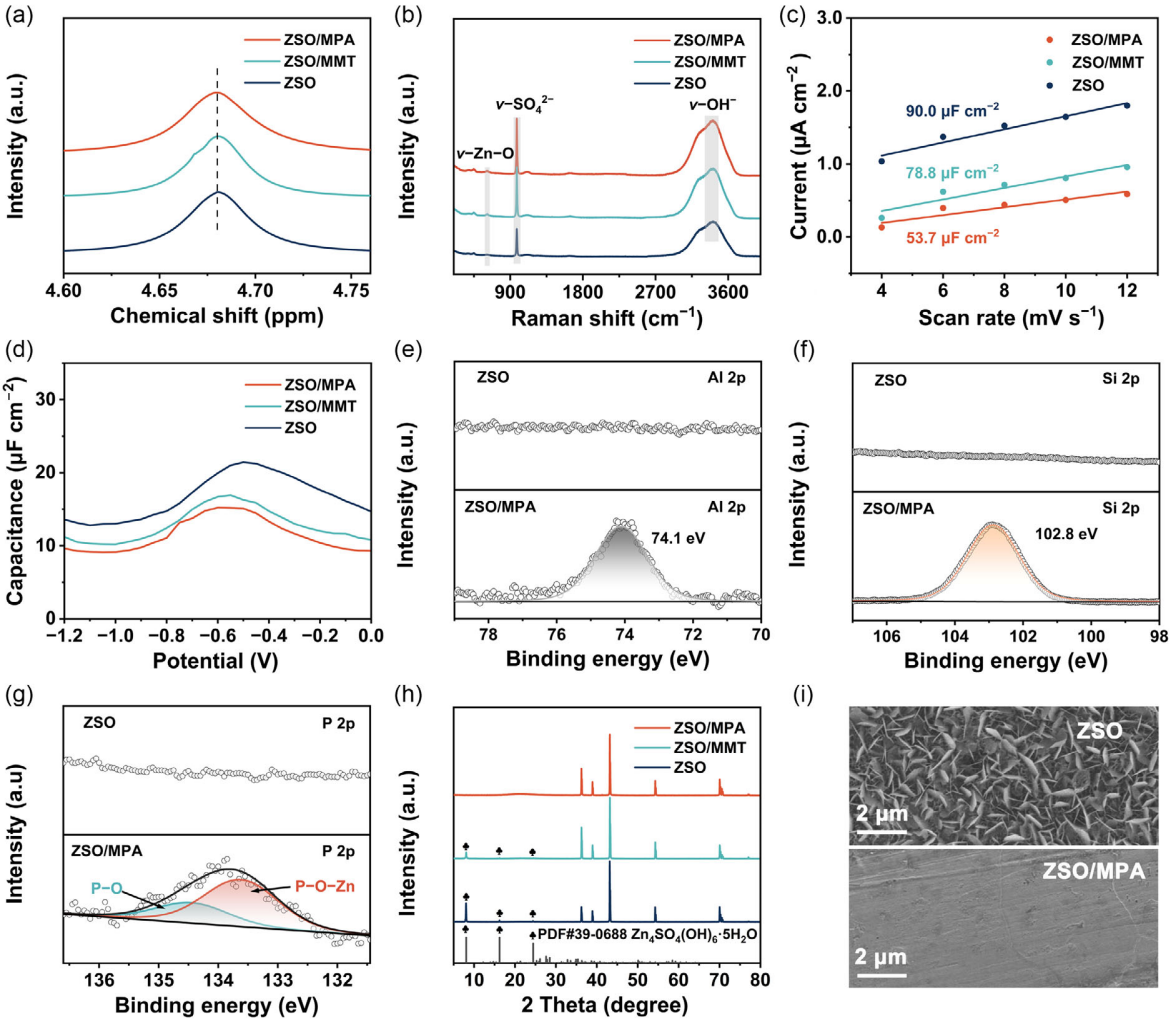
a) ^2^H NMR spectra of different electrolytes. b) Raman spectra of different electrolytes. c) EDL capacitance for cells with different electrolytes. d) Differential capacitance curves of Zn anodes with different electrolytes. The XPS spectra of e) P 2p, f) Si 2p, and g) Al 2p on the Zn anodes surface soaked in ZSO and ZSO/MPA electrolytes. h) XRD patterns for Zn electrodes after soaking 7 days in different electrolytes. i) SEM images of Zn electrodes after immersed in ZSO and ZSO/MPA electrolytes for 7 days.

To comprehensively investigate the influence of different electrolytes on Zn^2+^ transport kinetics, the transference number of Zn^2+^ (t_Zn_^2+^) was determined by combining chronoamperometry (CA) results with EIS measurements at initial and steady state (Figure 1d–f; Figure S9, Supporting Information). The t_Zn_^2+^ of the cells with the ZSO/MPA electrolyte achieves 0.80, significantly higher than that in the ZSO electrolyte (0.49) and the ZSO/MMT electrolyte (0.70), confirming the enhanced ionic sieving effect of the MPA‐based interface layer, which is attributed to the natural channel structure of the MMT and the elaborated microenvironmental regulation. Besides, the ZSO/MPA electrolyte exhibits a higher ionic conductivity of 31.9 mS cm^−1^, surpassing that of the ZSO/MMT electrolyte (26.8 mS cm^−1^) and the ZSO electrolyte (18.4 mS cm^−1^) (Figure 1g). The results demonstrate that improved ion diffusion and deposition kinetics in the ZSO/MPA electrolyte can effectively promote even Zn deposition and inhibit dendrite growth and the parasitic side reactions.^[^ ^33^, ^34^ ^]^ Nuclear magnetic resonance (NMR) spectroscopy and Raman spectroscopy were employed to probe the modulation of Zn^2+^ solvation environments induced by MPA additive within the electrolyte system. The ^2^H peak of D_2_O in the ZSO electrolyte is located at 4.68 ppm, while the peak positions of the ZSO/MMT electrolyte and the ZSO/MPA electrolyte remain unchanged, indicating that there is no obvious interaction between MPA and [Zn(D_2_O)_6_]^2+^, and thus the original hydrogen bonding network and solvation equilibrium state remains undisturbed (**Figure** **2**a).^[^ ^35^ ^]^ The characteristic peaks in the Raman spectra, attributed to Zn−O, SO_4_^2−^, and OH^−^, were observed in all three electrolytes without significant shifts (Figure 2b), suggesting that these low‐concentration additives have a negligible influence on the solvation structure of Zn^2+^,^[^ ^36^, ^37^ ^]^ corroborating the previously discussed NMR results. These results demonstrate that neither MMT nor MPA additives affect the solvation structure of Zn^2+^, thereby preserving the interaction between the Zn^2+^ core and the outer layer OSO_3_^2−^.

To further corroborate the adsorption behavior of MPA on the Zn metal surface, the double‐layer capacitance (EDLC) and differential capacitance curves were collected and analyzed (Figure 2c,d; Figure S10, Supporting Information). As expected, the MPA‐modified electrolyte reveals a lower measured EDLC capacitance value (53.7 μF cm^−2^) than ZSO (90.0 μF cm^−2^) and ZSO/MMT (78.8 μF cm^−2^). The differential capacitance in MPA‐modified electrolyte was also significantly lower compared to other electrolytes, further confirming that the MPA additive exhibits uniform adsorption behavior on the Zn metal surface.^[^ ^38^ ^]^ This is crucial for guiding the dense and uniform electroplating of Zn and enhancing the electrochemical stability of AZBs.^[^ ^39^, ^40^ ^]^ To demonstrate the positive effect of the MPA, the Zn foil was immersed in various electrolytes. The deconvoluted X‐ray photoelectron spectroscopy (XPS) spectra of the Zn metal with MPA additives not only exhibit diffraction peaks of MMT such as Al 2p (74.1 eV), Si 2p (102.8 eV),^[^ ^41^ ^]^ but also show the P–O–Zn peak (133.5 eV) (Figure 2e–g), indicating that the MPA nanosheets were adsorbed on the Zn metal surface through phosphate groups during the immersion process.^[^ ^42^ ^]^ The characteristic byproduct peaks of Zn_4_SO_4_(OH)_6_·5H_2_O (ZHS) were observed. XRD was observed for Zn metal immersed in both the ZSO and ZSO/MMT electrolytes, indicating the spontaneous corrosion of Zn anode in the electrolyte (Figure 2h). Conversely, no byproduct signals indicative of side reactions were detected for the Zn metal after introduction of the MPA additive, suggesting that the MPA additive effectively suppresses the occurrence of side reactions. The corrosion morphology of Zn electrodes in various electrolytes was investigated (Figure 2i; Figure S11, Supporting Information). Obviously, the Zn metal surface immersed in the ZSO electrolyte was covered with large amounts of flaky products. In contrast, an even, smooth, and compact surface morphology of Zn anode was observed after being soaked in MPA‐modified electrolyte, suggesting the superior ability of the protection layer derived from MPA additive in inhibiting side reactions.

**Figure 3:**
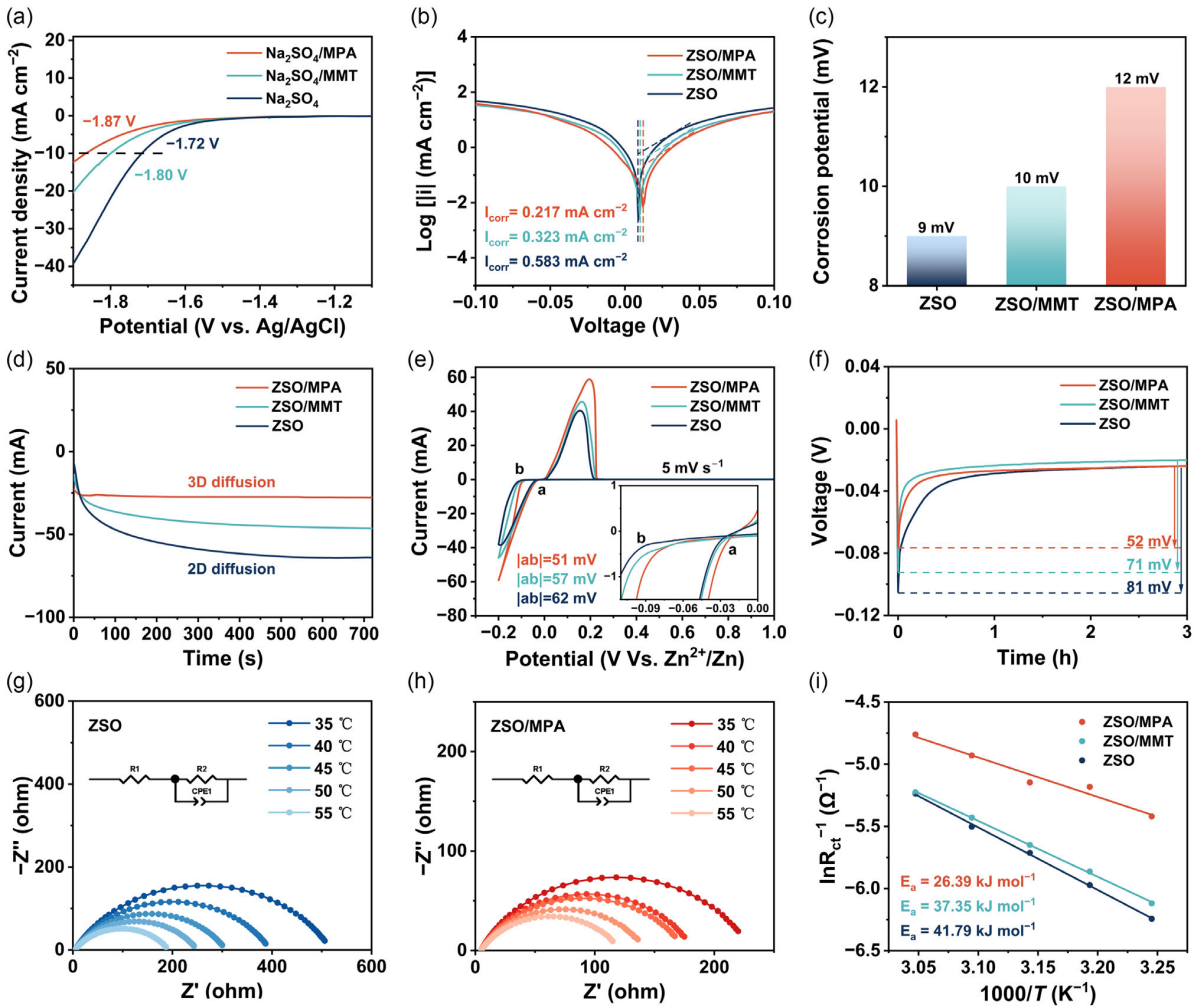
a) Linear polarization curves of Zn anodes with different electrolytes using a three‐electrode cell. b) Tafel curves of Zn anodes with different electrolytes. c) Corrosion potential of Zn anodes in different electrolytes. d) CA curves of the symmetric cells with different electrolytes. e) CV curves of Zn anodes in different electrolytes. f) Voltage‐capacity profiles of Zn anodes with various electrolytes at 1 mA cm^−2^. Nyquist plots of Zn||Zn symmetrical cells in g) ZSO and h) ZSO/MPA electrolytes at different temperatures. i) Comparison of Arrhenius curves of different electrolytes.

To precisely assess the inhibitory effects of diverse additives on the hydrogen HER and corrosion, the LSV test of Zn anodes in 1 M Na_2_SO_4_ electrolyte was conducted (**Figure** **3**a).^[^ ^43^ ^]^ An improved HER overpotential is observed on the Zn anode within the MPA additive, indicating inhibition of the water decomposition.^[^ ^44^ ^]^ Furthermore, the Zn anode with ZSO/MPA electrolyte displays a higher corrosion potential (12 mV) and lower corrosion current (0.217 mA cm^−2^) compared to the Zn anode (9 mV; 0.583 mA cm^−2^) in ZSO electrolyte, confirming its superior anticorrosion properties and suppression of side reactions in ZSO/MPA electrolyte (Figure 3b,c).^[^ ^45^ ^]^ The nucleation behavior of Zn^2+^ in various electrolytes was elucidated in Figure 3d. In ZSO electrolyte, the Zn deposition currents progressively increase during the initial 600 s, suggesting a long, disordered 2D nucleation and diffusion behavior relevant to severe dendrite growth.^[^ ^46^ ^]^ By contrast, the 2D diffusion process occurs within 50 s after the introduction of MPA additives, followed by a steady and constant 3D diffusion process throughout the plating process, suggesting an even, compact, and smooth Zn deposition in ZSO/MPA electrolyte. Furthermore, as shown in Figure 3e, the higher peak current of Zn anode with MPA additives is observed during cycling, indicating the improved Zn deposition capacity. The superior performance is attributed to the reduced nucleation overpotential of Zn, which is further confirmed in Figure 3f. The effect of different electrolytes on Zn migration was evaluated using impedance measurement at various temperatures (Figure 3g,h; Figure S12 and Table S2, Supporting Information). After the introduction of MPA additives, the activation energy (*E*_a_) of the Zn anode decreases from 41.79 kJ mol^−1^ (ZSO) to 26.39 kJ mol^−1^ in (ZSO/MPA) (Figure 3i), suggesting enhanced desolvation kinetics.^[^ ^47^, ^48^, ^49^ ^]^

**Figure 4:**
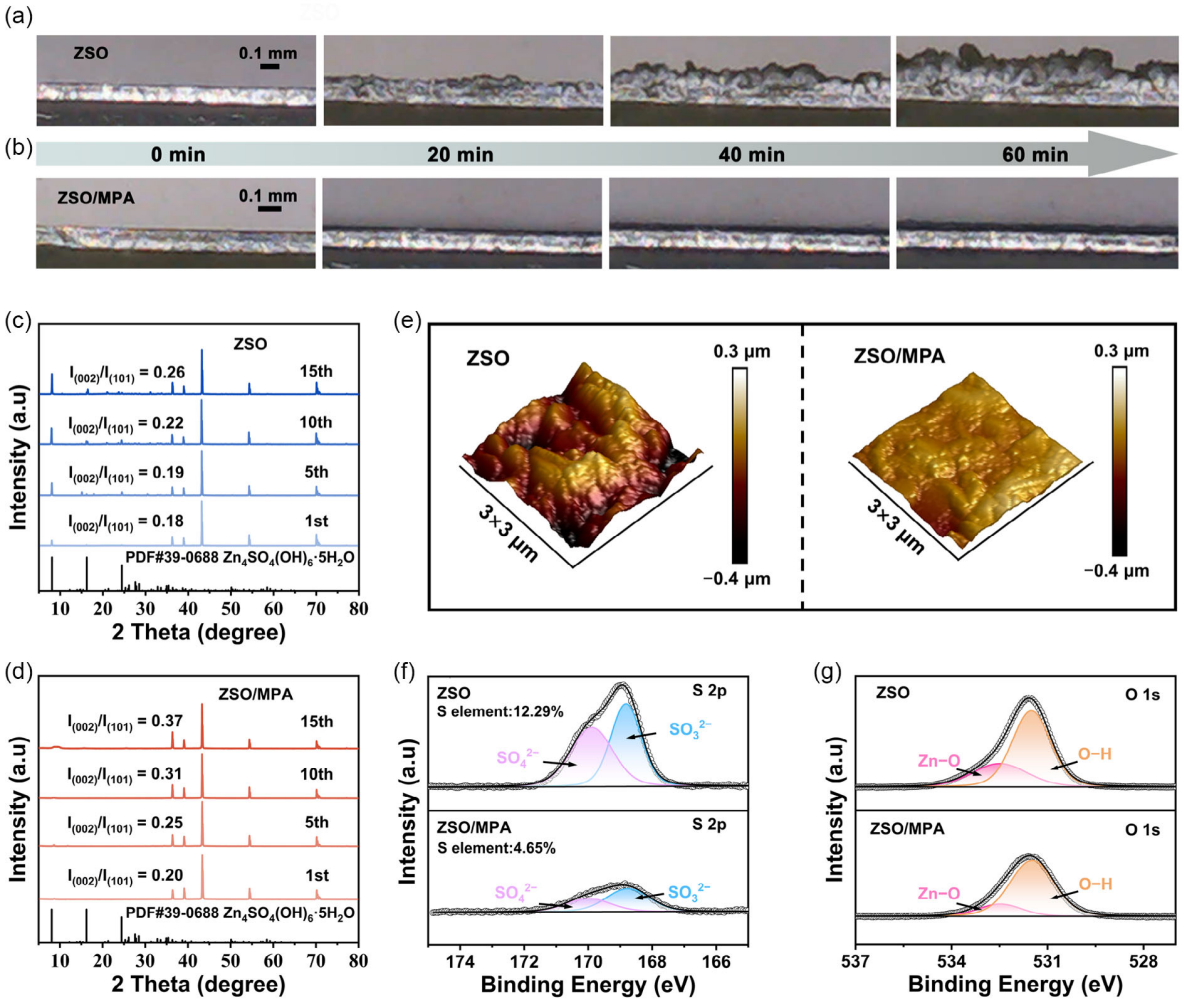
In situ optical microscopy observation of Zn deposition in a) ZSO and b) ZSO/MPA electrolytes. XRD patterns of Zn anodes after different cycles in c) ZSO and d) ZSO/MPA electrolytes. e) AFM images of the cycled Zn anodes surface with different electrolytes. f) XPS spectra of S 2p of Zn anodes after cycling in ZSO and ZSO/MPA electrolytes. g) XPS spectra of O 1s of Zn anodes after cycling in ZSO and ZSO/MPA electrolytes.

To directly monitor the regulatory effects of various electrolytes on the stability of the Zn anodes, in situ optical microscopy was utilized. During the initial 20 min of electroplating, Zn^2+^ in the ZSO electrolyte exhibited pronounced nonuniform deposition, leading to dendrite formation. After 60 min of plating, these dendrites grew uncontrollably (**Figure** **4**a). While the ZSO/MMT electrolyte partially mitigates the side reactions and dendrite growth, the heterogeneous MMT adsorption layer causes spatially inconsistent Zn^2+^ deposition, ultimately resulting in a thick and irregular deposition layer (Figure S13, Supporting Information). Notably, Zn^2+^ deposition in the ZSO/MPA electrolyte was flat and uniform, with no observable bubbles or dendrites, thereby intuitively demonstrating the superior performance of MPA in regulating Zn^2+^ deposition behavior and suppressing the side reactions (Figure 4b). The XRD patterns confirmed that during the prolonged cycling processes, in ZSO electrolyte, the intensity ratio of Zn (002) to Zn (101) (denoted as I_(002)_/I_(101)_) remained nearly constant (Figure 4c). On the contrary, the ratio of *I*_(002)_/*I*_(101)_ increased significantly within ZSO/MPA electrolyte (Figure 4d). Besides, the peaks at 8.1°, 16.2°, and 24.4°, corresponding to the ZHS, were observed on the Zn anode with ZSO electrolyte, but were absent on the anode with ZSO/MPA electrolyte, indicating that the MPA‐based protection layer effectively restrains the formation of the ZHS byproducts during the stripping/plating process. This demonstrates that MPA adsorbed on the Zn anode can induce preferential deposition along the Zn (002) plane, effectively suppressing the formation of byproducts and ultimately achieving uniform Zn metal deposition.^[^ ^50^ ^]^ Furthermore, the cycled Zn anode surface displays uneven coloration, suggesting a coarse texture and the generation of Zn dendrites (Figure 4e; Figures S14 and S15, Supporting Information). In contrast, the Zn anode in the ZSO/MPA electrolyte presents a smooth morphology, devoid of any dendrites, demonstrating that the enhancement in Zn^2+^ plating kinetics is aided by the MPA‐based interphase layer. Furthermore, the composition of the electrodeposited Zn was systematically investigated using XPS (Figure 4f). The peaks at 168.7 and 169.9 eV in the S 2p spectrum are attributed to SO_3_^2−^ and SO_4_^2−^, respectively. The O 1s spectrum can be deconvoluted into peaks at 532.4 and 531.5 eV, corresponding to Zn—O and O—H bonds, respectively (Figure 4g). The presence of SO_4_^2−^ and OH^−^ indicates the formation of Zn_4_SO_4_(OH)_6_·xH_2_O on the bare Zn surface. Notably, Zn anode in ZSO/MPA electrolyte exhibits significantly reduced elemental sulfur content and a lower SO_4_^2−^/SO_3_^2−^ ratio after cycling, further validating the efficacy of MPA nanosheets in suppressing water‐induced parasitic reactions.^[^ ^51^ ^]^

**Figure 5:**
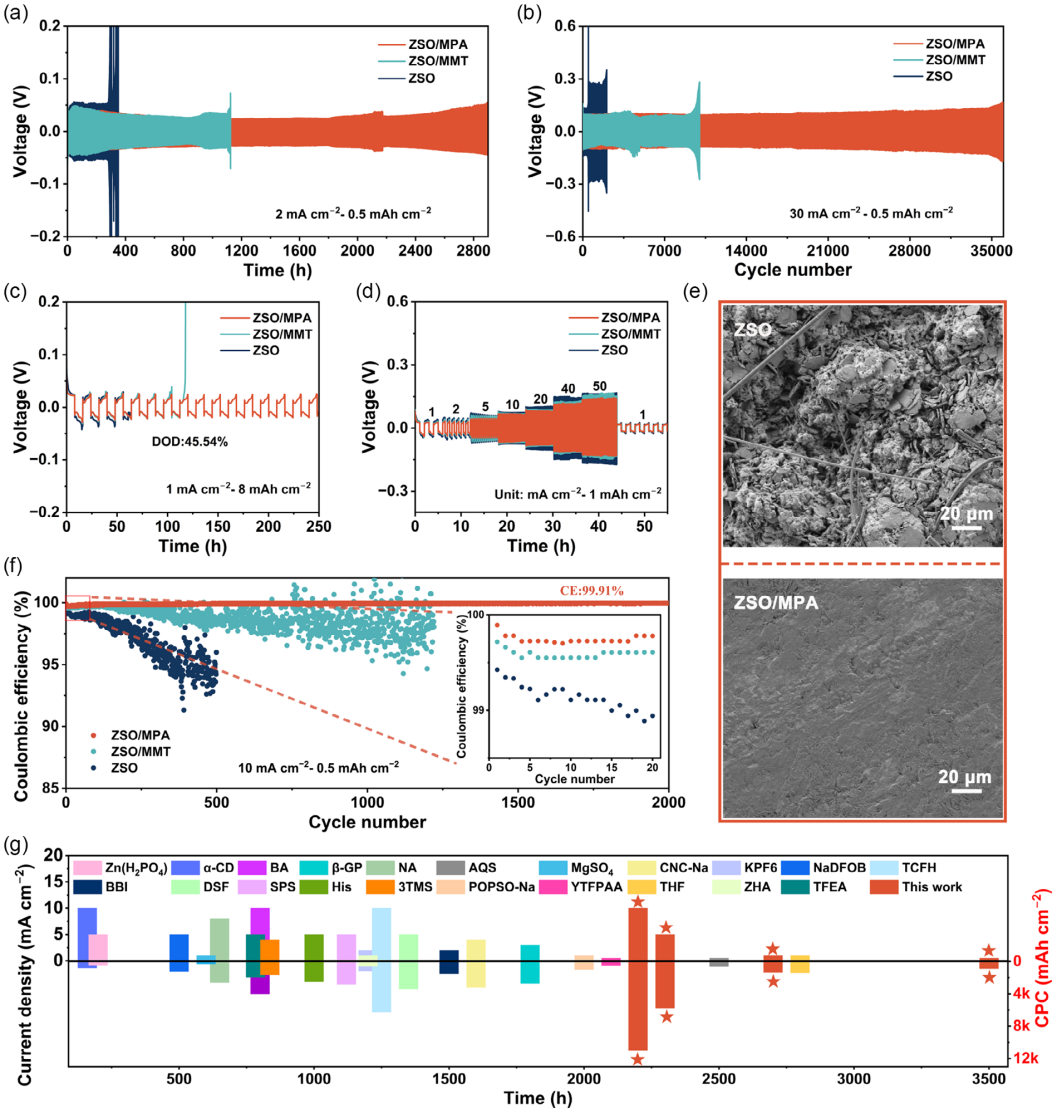
Cycling performance of Zn||Zn symmetric cells in various electrolytes at a) 2 mA cm^−2^, 0.5 mAh cm^−2^, b) 30 mA cm^−2^, 0.5 mAh cm^−2^. c) Zn||Zn symmetric cells in various electrolytes cycling with a DOD of 45.54%. d) Rate performance of Zn||Zn symmetric cells. e) SEM images of Zn anodes after 100 cycles in different electrolytes. f) The CE curves of Zn||Cu cells in different electrolytes at 10 mA cm^−2^ and 0.5 mAh cm^−2^ for the first 20 cycles (inset). g) Comparison of cyclic lifespans in recent reports.

To deeply evaluate the effect of the MPA additive on the electrochemical performance of the AZBs, the galvanostatic plating/stripping tests of the Zn||Zn symmetric cells were conducted at various current densities and areal capacities. In **Figure** **5**a, the symmetrical cell using ZSO/MPA electrolyte displays stable cycling stability of over 2800 h. Conversely, the symmetrical cells undergo premature failure due to a short‐circuiting at 280 h (ZSO) and 1123 h (ZSO/MMT), respectively, which can be attributed to severe parasitic side reactions and rampant Zn dendrite formation. The symmetrical cell with MPA additives also delivers significantly prolonged cycle lifetimes at various current densities (Figure S16, Supporting Information). Remarkably, under an ultrahigh current density of 30 mA cm^−2^, the Zn||Zn symmetric cell incorporating the ZSO/MPA electrolyte still exhibits exceptional cyclability, sustaining operation over 36 000 cycles while maintaining minimal voltage polarization. In sharp contrast, the cells experience abrupt failure at merely 480 cycles (ZSO) and 9950 cycles (ZSO/MMT), respectively (Figure 5b). Depth of discharge (DOD) is a crucial evaluation metric for battery systems, directly correlating with their achievable energy density and operational longevity under practical high‐energy‐demand scenarios.^[^ ^52^ ^]^ Under rigorous deep‐discharge testing conditions (DOD_Zn_ = 45.54%), the Zn anode in ZSO electrolyte delivers catastrophic failure within 60 h. Remarkably, the Zn anode with MPA additives still maintains stable operation for over 250 h under identical conditions (Figure 5c). The superior performance of the ZSO/MPA electrolyte underscores its outstanding reduction in active Zn loss throughout the entire cycling. Figure 5d systematically compares cycling stability of the symmetric cells employing the three electrolytes at various current densities of 1, 2, 5, 10, 20, 40, and 50 mA cm^−2^. The cell with ZSO/MPA electrolyte delivers significantly decreased voltage hysteresis compared to the cells with the ZSO/MMT and ZSO electrolytes, reflecting enhanced Zn deposition kinetics (Table S3, Supporting Information).^[^ ^53^ ^]^ In Figure S17, Supporting Information, the Zn anode with MPA additives delivers an increased exchange current density (*I*_0_), further confirming that the introduction of MPA additive can effectively enhance Zn^2+^ diffusion kinetics.^[^ ^54^ ^]^

The top‐view SEM image of the cycled Zn anode in the ZSO electrolyte exhibits a multitude of irregular flake‐like Zn deposits and randomly distributed dendritic Zn, suggesting rather nonuniform Zn^2+^ deposition and continuous side reactions between Zn and the electrolyte. In sharp contrast, the cycled Zn anode surface in ZSO/MPA electrolyte appears denser and smoother, demonstrating the uniform deposition of Zn^2+^ (Figure 5e; Figure S18, Supporting Information). In contrast, dendrites and byproducts were still formed on the Zn anode surface within ZSO/MMT electrolyte, resulting from the weak interaction between the metallic anode and MMT nanosheets, and thus induced an unstable interface layer (Figure S19, Supporting Information). CE is a crucial metric for assessing the cycling reversibility of Zn.^[^ ^55^ ^]^ In ZSO electrolyte, the Zn||Cu asymmetric cell displays an initial CE of 99.4% and a short‐circuit after 200 cycles, which is attributed to the severe Zn dendrite growth and the persistent formation of dead Zn.^[^ ^56^ ^]^ After the introduction of MMT additives, the initial CE increased from 99.4% to 99.7%, and the cycle life extended to 500 cycles, indicating that MMT additives can, to a certain extent, enhance the reversibility of Zn deposition. In contrast, the Zn anode demonstrates superior cycling stability over 2000 cycle with a high average CE of 99.9%, accompanied by a minimal hysteresis (Δ*V* ≈ 82.4 mV) throughout the Zn plating/stripping processes (Figure 5f; Figure S20, Supporting Information), which is attributed to the effect of the MPA‐derived interfacial layer on inhibiting the Zn dendrite formation, HER, and promoting the even and reversible Zn deposition. The significantly improved cycling stability and CE can be attributed to the PA‐MMT interfacial layer, which effectively suppresses side reactions between the electrolyte and the Zn metal anode while simultaneously homogenizing the Zn ion flux to enable dendrite‐free Zn deposition. These results directly validate the electrolyte's ability to maintain electrode–electrolyte interfacial stability while suppressing dendritic Zn formation and side reactions. Moreover, the parameter of DOD, the cumulative plated capacity (CPC), and cycle life are more objective and reliable indicators for evaluating the overall performance of Zn anodes.^[^ ^52^ ^]^ Figure 5g presents a comparison with previous research results regarding lifespan, current density, and CPC, further confirming that the ZSO/MPA electrolyte significantly enhances the electrochemical performance of the Zn anodes (Table S4, Supporting Information).

**Figure 6:**
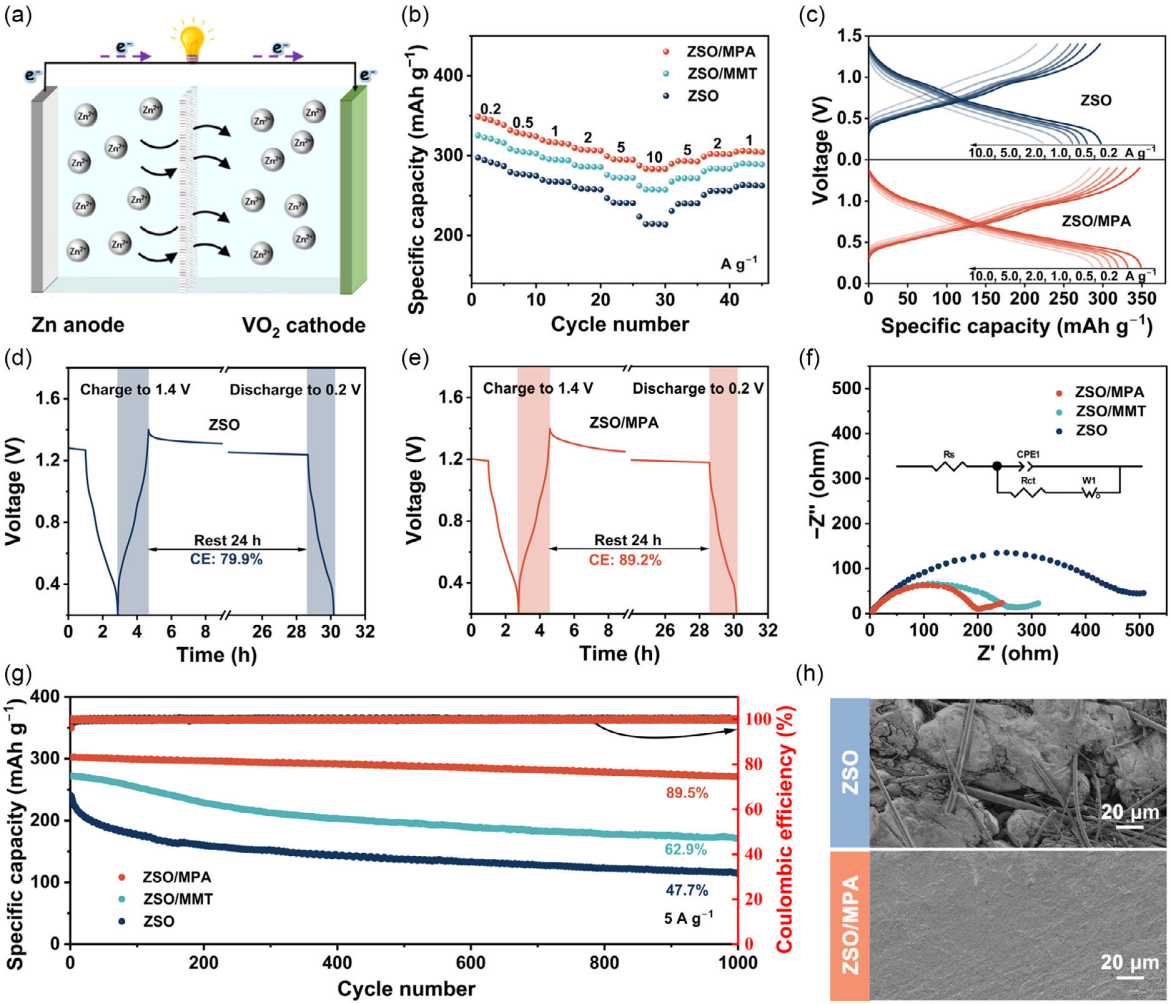
a) A schematic illustration of Zn||VO_2_ full cell. b) Rate performance of Zn||VO_2_ full cells within different electrolytes and c) the corresponding charge and discharge profiles. Self‐discharge behavior of full cells within d) ZSO and e) ZSO/MPA electrolytes. f) Nyquist plots of the full cells within different electrolytes. g) Cycling stability of full cells at 5 A g^−1^. h) SEM images of full cells at various electrolytes after 500 cycles at 5 A g^−1^.

To further demonstrate the compatibility and practical applications of the ZSO/MPA electrolyte, a Zn||VO_2_ full cell was assembled by coupling the Zn metal anode with the VO_2_ cathode (**Figure** **6**a; Figures S21 and S22, Supporting Information). Zn||VO_2_ full cells retain similar redox characteristics within various electrolytes, suggesting that MPA nanosheet additives do not alter the intrinsic electrochemical mechanism of the VO_2_ cathode (Figure S23, Supporting Information).^[^ ^57^ ^]^ The full cell within the ZSO/MPA electrolyte exhibits superior rate capability at various current densities of 0.2, 0.5, 1, 2, 5, and 10 A g^−1^ and delivers specific capacities that surpass those of the full cells within the ZSO and the ZSO/MMT electrolytes (Figure 6b). The full cell within ZSO/MPA electrolyte exhibits minimal voltage polarization compared to that of the full cells within ZSO and ZSO/MMT electrolytes (Figure 6c; Figure S24, Supporting Information), suggesting enhanced stabilization of the electrode–electrolyte interface and improved reaction kinetics. The full cell within the ZSO/MPA electrolyte also exhibits enhanced calendar aging resistance, maintaining 89.2% of its initial CE after 24 h of storage, which is higher than that of the cell within the ZSO/MMT electrolyte (85.6%) and the cell within the ZSO electrolyte (79.9%) (Figure 6d,e; Figure S25, Supporting Information). EIS analysis reveals that the full cell with MPA additives displays a lower charge transfer resistance (*R*_ct_) of 195 Ω compared to full cells with ZSO/MMT electrolyte (245 Ω) and ZSO electrolyte (480 Ω) (Figure 6f). Additionally, the Zn^2+^ diffusion coefficient (*D*_Zn_) for the full cell with MPA additives is 5.36 × 10^−12^ cm^2^ s^−1^, significantly higher than that in full cells with ZSO (4.70 × 10^−13^ cm^2^ s^−1^) and ZSO/MMT (7.40 × 10^−13^ cm^2^ s^−1^) electrolytes. The results confirmed that MPA additives effectively promote the diffusion of Zn^2+^ and accelerate charge transfer (Figure S26, Supporting Information). In Figure 6g, the full cell employing ZSO/MPA electrolyte demonstrates exceptional cycling stability, achieving a capacity of 271 mAh g^−1^, with a capacity retention of 89.5%, significantly outperforming full cells within the ZSO electrolyte (47.7%) and the ZSO/MMT electrolyte (62.9%). In Figure 6h, the Zn deposition morphology of cycled Zn anode within ZSO electrolyte exhibits loose structures with numerous dendrites and byproducts, ascribed to nonuniform Zn deposition and water‐induced parasitic reactions. While the cycled Zn anode with MMT additives demonstrates improved surface planarization to some extent, it still suffers from massive Zn dendrite growth (Figure S27, Supporting Information). Remarkably, the ZSO/MPA electrolyte facilitates dendrite‐free Zn deposition by homogenizing the ion flux and constructing a stable interfacial layer. These results further demonstrate the dual functionality of the modified electrolyte in suppressing both dendritic Zn growth and water‐induced parasitic reactions.

## 3 Conclusion

In summary, a microenvironment‐controlled additive strategy was proposed, involving the development of PA‐functionalized MMT nanosheets as electrolyte additives for AZBs. The MPA‐based interface layer enables hierarchical regulation of Zn^2+^ deposition dynamics through spontaneous interfacial self‐assembly, which is driven by chemisorption interactions. Systematic experimental characterizations demonstrate that the MPA‐derived interfacial protective layer exhibits two primary functions: 1) effective suppression of electrolyte‐induced corrosion via preferential adsorption onto electrochemically active sites; 2) homogenization and acceleration of Zn^2+^ flux via intrinsic ion transport pathways within the crystal structure and enhanced interfacial zincophilicity enabled by the tailored microenvironment. Benefiting from these advantages, the Zn||Zn symmetric cells demonstrate exceptional stability, maintaining stable operation for over 2800 h at 2 mA cm^−2^ with an areal capacity of 0.5 mAh cm^−2^. Remarkably, even under an ultrahigh current density of 30 mA cm^−2^, the Zn anode achieves prolonged cycling over 30 000 cycles. Moreover, the Zn||Cu half cell exhibits superior electrochemical performance with an average CE of 99.9%. Furthermore, the assembled Zn||VO_2_ full cell delivers outstanding long‐term cycling stability and capacity retention (89.5%). Our study provides innovative strategies and methodologies for the interface engineering and electrolyte design for the Zn anodes, offering a new perspective for developing high‐performance and stable energy storage systems.

## 4 Experimental Section

4.1

4.1.1

##### Materials

Zn foils (100 and 30 μm, 99.99%) and PA solution (70% in H_2_O) were purchased from Shanghai Aladdin Biochemical Technology Co., Ltd. Zinc sulfate (ZnSO_4_·7H_2_O, 99.99%), MMT, ethylene glycol (99.5%), and absolute ethyl alcohol (99.7%) were purchased from Sinopharm Chemical Reagent Co., Ltd. NH_4_VO_3_ (99%) was purchased from Macklin Chemical Reagent Co., Ltd. DI water (18.25 MΩ cm) used in this work was acquired through ultrapure water purifiers (Milli‐Q). All chemicals and materials were used without further purification.

##### Preparation of ZSO/MMT and ZSO/MPA Electrolytes

MMT nanosheets were prepared through a liquid‐phase exfoliation process. Specifically, MMT (5 g) was dispersed in DI water (1 L) under continuous magnetic stirring for 7 days. The suspension was centrifuged twice at 3000 rpm to remove unexfoliated MMT aggregates, yielding a stable colloidal supernatant that remained aggregation‐free for over 7 days. The supernatant was lyophilized to obtain MMT nanosheets. For the synthesis of MPA nanosheets, PA (1 g) was dissolved in deionized water (50 mL) to form a homogeneous solution. This PA solution was titrated into predetermined volumes of MMT colloidal suspension under continuous stirring at 25 °C for 24 h. The resulting mixture was vacuum filtered through a cellulose ester membrane (0.22 μm), followed by sequential rinsing with deionized water to eliminate residual PA. The samples were lyophilized to obtain MPA nanosheets. The ZSO/MMT and ZSO/MPA electrolytes were formulated by dispersing MMT nanosheets and MPA composite nanosheets, respectively, into ZnSO_4_ aqueous solution (2 mol L^−1^).

##### Preparation of VO_*2*_ *Electrode*

Vanadium dioxide (VO_2_) nanobelts were synthesized via a hydrothermal route. Specifically, ammonium metavanadate (NH_4_VO_3_) (0.7 g) and ethylene glycol (20 mL) were dissolved in deionized water (40 mL) under continuous magnetic stirring. The pH was adjusted to ≈1.5 by dropwise addition of HCl (2 mol L^−1^). The homogeneous solution was transferred into a polytetrafluoroethylene‐lined autoclave (100 mL) and maintained at 180 °C for 6 h. The resulting dark precipitates were collected, washed sequentially with deionized water and ethanol, and vacuum dried at 80 °C.^[^ ^1^ ^]^ For cathode fabrication, a slurry containing VO_2_ nanobelts, acetylene black, and polyvinylidene fluoride binder (7:2:1 mass ratio) was homogenized in N‐methyl‐2‐pyrrolidone solvent. The slurry was uniformly coated onto titanium foil (current collector) and dried at 60 °C for 12 h under vacuum. The active material mass loading was controlled at 1‐2 mg cm^−2^.

##### Electrochemical Measurements

Zn||Zn symmetric coin cells (CR2032) were fabricated by sandwiching a glass fiber separator impregnated with various electrolytes between two 12 mm‐diameter zinc foils. The Zn||Cu asymmetric cells and Zn||VO_2_ full cells were assembled following identical procedures, with substitution of one zinc electrode by a copper foil or presynthesized VO_2_ cathode, respectively. Electrochemical characterizations, including cyclic voltammetry (CV) (0.2–1.4 V), EIS (10^5^–10^−1^ Hz), Tafel polarization, and CA tests, were conducted on a CHI 760 E electrochemical workstation (Chenhua Instrument Company, Shanghai, China). LSV measurements employed a three‐electrode configuration with zinc foil (working electrode), platinum foil (counter electrode), and Ag/AgCl (reference electrode). Galvanostatic charge–discharge cycling, CE evaluation, self‐discharge tests, and long‐term cycling assessments were performed using a Neware battery testing system (CT‐3008, China).

##### Characterization

The electrolyte was studied by Raman spectrometer (inVia‐Reflex) and NMR spectrometer (ECZ600R, JEOL Resonance Inc.) XRD (MAP18AHF, Kα‐radiation, at 40 kV and 20 mA) with copper target as radiation source was used to study the phase and crystal structure of the sample. The surface morphology of the samples was characterized by field‐emission scanning electron microscopy (S‐4800). The chemical composition of Zn foil was characterized by XPS of ESCA Lab MK II.

The cation migration number ($t_{{\text{Zn}}^{2+}}$) is computed as outlined below

(1) $$t_{{\text{Zn}}^{2+}}\;=\;\frac{\Delta V/I_{0}-R_{0}}{\Delta V/I_{s}-R_{s}}$$

in polarization measurements, *I*_0_ represents the initial state current, while *I*_s_ denotes the steady state current. The resistance values *R*_0_ and *R*_s_ are obtained from EIS measurements conducted before and after polarization, respectively. The applied polarization potential is denoted as Δ*V* (10 mV).

The calculation of activation energy ($E_{a}$) was done based on EIS analysis at different temperatures. The $E_{a}$ can be quantitatively calculated by the following Arrhenius equation

(2) $$\frac{1}{R_{\text{ct}}}=\;A\;\exp\;\left(\frac{-E_{a}}{RT}\right)$$

where *R*_ct_, *E*_a_, *A*, *R*, and *T* denote the charge transfer resistance, activation energy, preexponential factor, universal gas constant, and absolute temperature (K), respectively.

The ionic conductivity ($\sigma_{{\text{Zn}}^{2+}}$) was calculated according to the following equation^[^ ^58^ ^]^

(3) $$\sigma_{{\text{Zn}}^{\text{2+}}}\;=\;\frac{L}{R\cdot A}$$

where *R* is the series resistance, *L* is the thickness of the separator between stainless steel electrodes (675 μm), and *A* is the total surface area of the stainless steel electrode (2.01 cm^2^).

The EDL capacitance (*C*) was calculated according to the following equation^[^ ^59^ ^]^

(4) $$C\;=\;\frac{i_{c}}{v}$$

where $i_{c}$ represents the capacitive current. Here, $i_{c}$ was defined as ($i_{0v^{+}}-i_{0v^{-}}$)/2, meaning half value of the current difference during forward scan and negative scan at 0 V. The scan rates of CV tests are denoted by *v*. The corresponding CV curves were obtained by scanning between −15 and 15 mV using Zn||Zn symmetric cells.

The Zn^2+^ diffusion coefficient (*D*) was calculated according to the following equation

(5) $$D=\frac{1}{2}{\left(\frac{RT}{nAF^{2}C\sigma }\right)}^{2}$$

where *R* is the gas constant, *T* is the absolute temperature, *A* is the surface area of the electrode, *n* is the number of electrons per molecule during oxidization, *F* is the Faraday constant, *C* is the concentration of Zn^2+^, and *σ* is the Warburg factor.

## Supporting Information

Supporting Information is available from the Wiley Online Library or from the author.

## Conflict of Interest

The authors declare no conflict of interest.

## Supporting information

Supplementary Material

## Data Availability

The data that support the findings of this study are available from the corresponding author upon reasonable request.
