# Supplementary material for: Montmorillonite Interfacial Chemistry Regulation on Homogeneous Zn Deposition: A Microenvironment‐Controlled Additive Strategy for Sustainable Zinc Metal Anodes
Source: Small Sci. 2025 Oct 16;5(12):e202500377. doi: 10.1002/smsc.202500377 (PMC12697821; doi:10.1002/smsc.202500377)
Supplement: Supplementary file 1 — Supplementary Material [file SMSC-5-e202500377-s001.pdf]

Supporting Information

Montmorillonite Interfacial Chemistry Regulation on Homogeneous Zn Deposition: A Microenvironment-Controlled Additive Strategy for Sustainable Zinc Metal Anodes

Hailong Xuan[1,#], Xiaolong Cheng[1,#], Yu Yao[3], Yihong Gao[2], Pengcheng Shi[1], Fangzhi Huang[2,], Yu Jiang[1,*], Yan Yu[3,*]*

[1] *School of Materials Science and Engineering, Anhui University, Hefei, 230601, China. E-mail:* jiangyu21@ahu.edu.cn.

[2] *School of Chemistry and Chemical Engineering, Anhui University, Hefei 230601, China. E-mail: hfz@ahu.edu.cn.*

[3] *Hefei National Research Center for Physical Sciences at the Microscale, Department of Materials Science and Engineering, University of Science and Technology of China, Hefei, Anhui 230026, China. Email: yanyumse@ustc.edu.cn.*

[#] *These authors contributed equally to this work.*

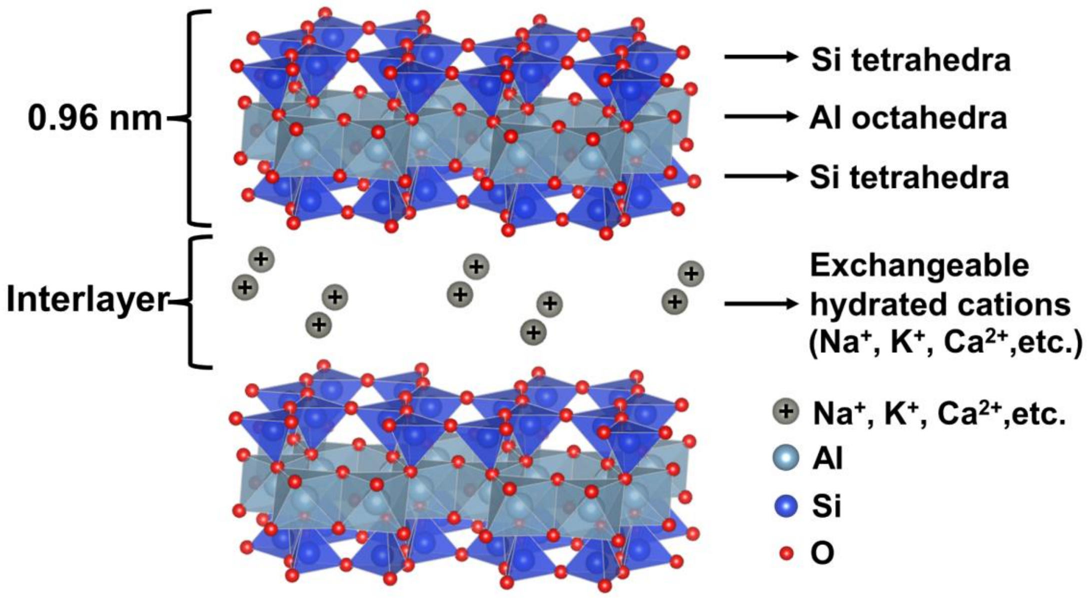

Figure S1. The Schematic structure of montmorillonite.

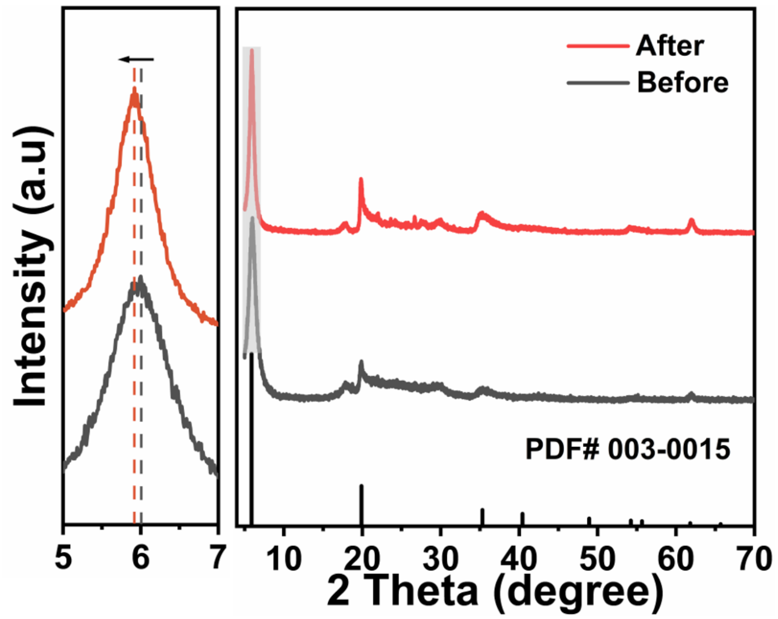

Figure S2. The XRD patterns of MMT before stripping and MMT after stripping.

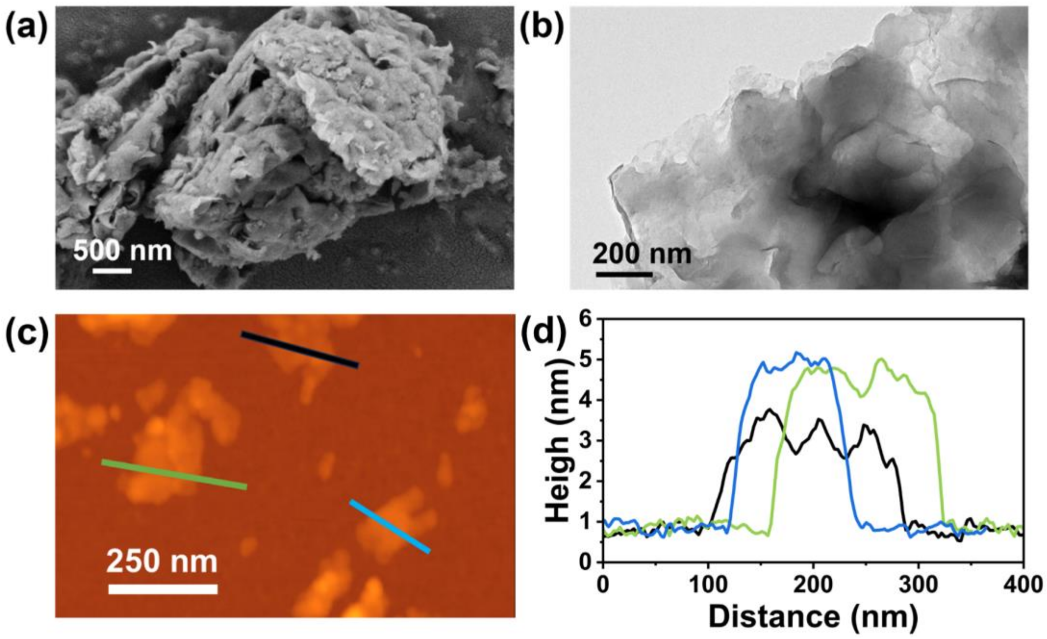

Figure S3. (a) The SEM image of MMT before stripping. (b) The TEM image of MMT after stripping. (c) AFM topography image and (d) the corresponding height profile of exfoliated MMT nanosheets.

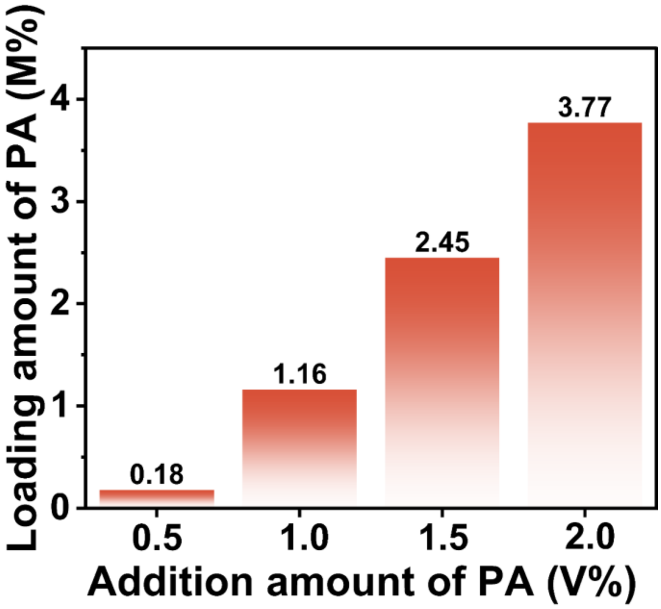

Figure S4. The inductively coupled plasma (ICP) quantitative analysis of P element on MPA.

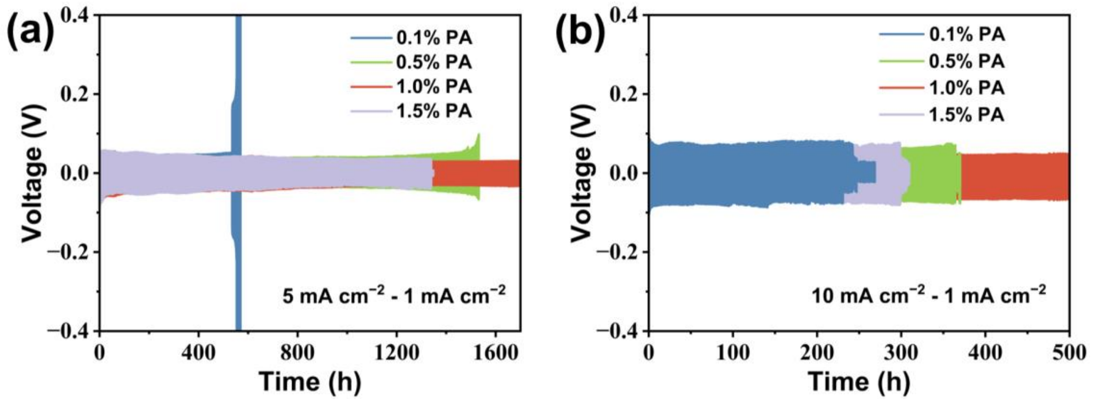

Figure S5. Long-term cycling performance of Zn||Zn symmetric cells with different concentrations of phytic acid modified MMT additives at (a) 5 mA cm^{-2} and 1 mAh cm^{-2}, (b) 10 mA cm^{-2} and 1 mAh cm^{-2}.

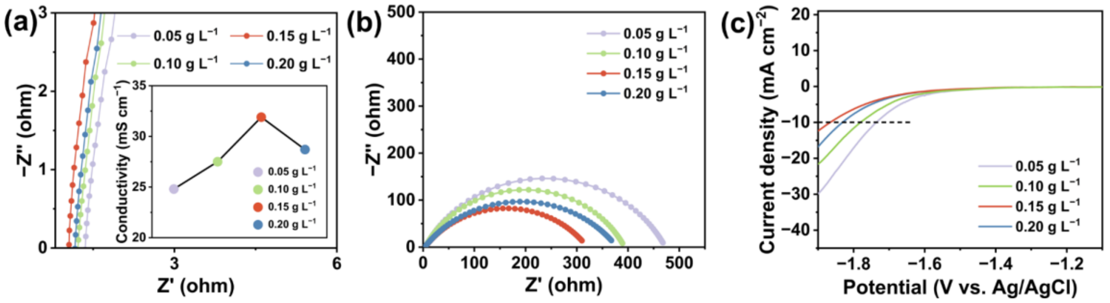

Figure S6. Electrochemical performance of electrolytes with varying MPA concentrations: (a)Ionic conductivity, (b) Nyquist plots, and (c) Linear sweep voltammetry under the three-electrode system.

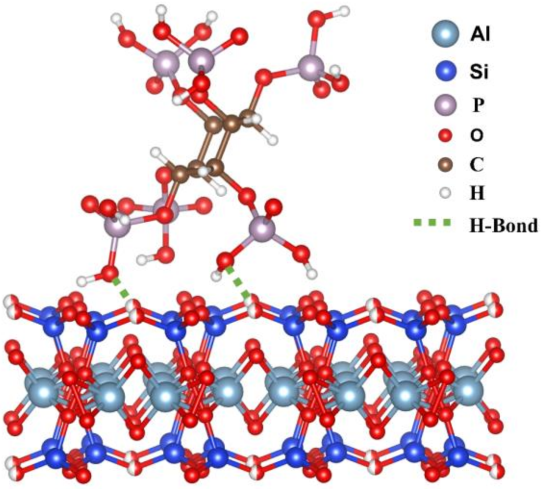

Figure S7. The schematic illustration of phytic acid (PA)-functionalized montmorillonite (MPA) nanosheets. The MPA nanosheets can spontaneously self-assemble onto the Zn anode surface due to the strong adsorption between PA groups and Zn metal, forming a protective layer that enables multi-level regulation of the Zn^{2+} deposition process.

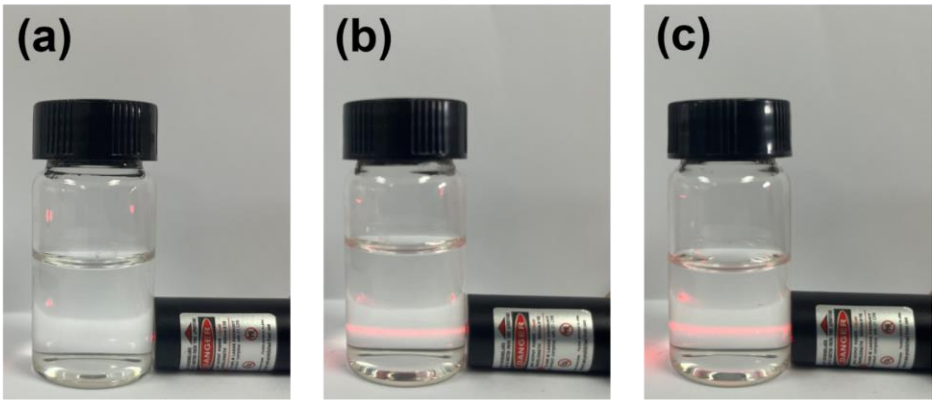

Figure S8. Digital images of (a) ZSO, (b) ZSO/MMT, and (c) ZSO/MPA electrolytes for the Tyndall effect.

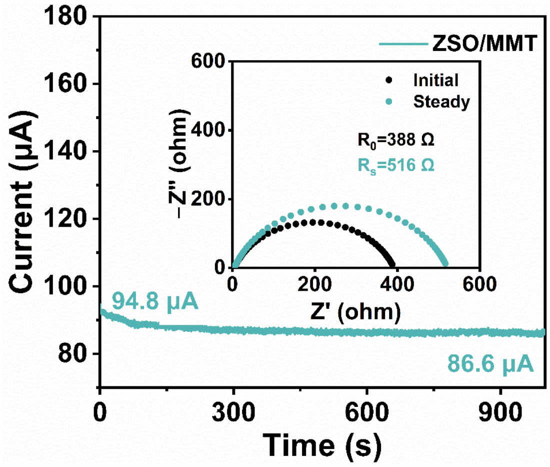

Figure S9. The chronoamperometry (CA) curves of Zn||Zn symmetric cells and corresponding Nyquist plots within ZSO/MMT electrolytes at the initial and steady states.

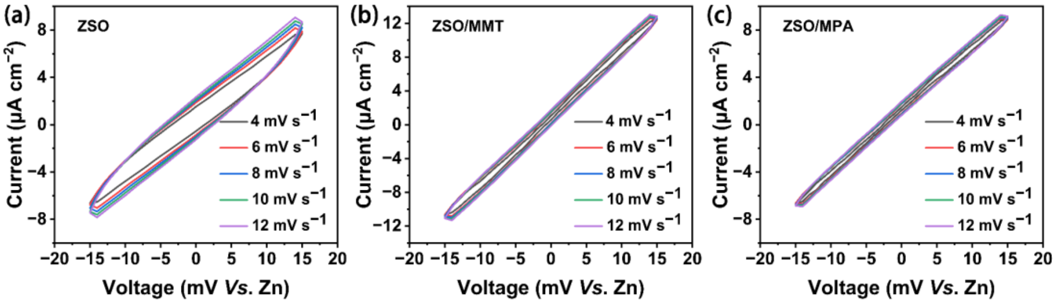

Figure S10. CV curves of Zn||Zn symmetrical cells in a voltage range of –15~15 mV under various scanning rates in (a) ZSO, (b)ZSO/MMT, and (c) ZSO/MPA electrolytes.

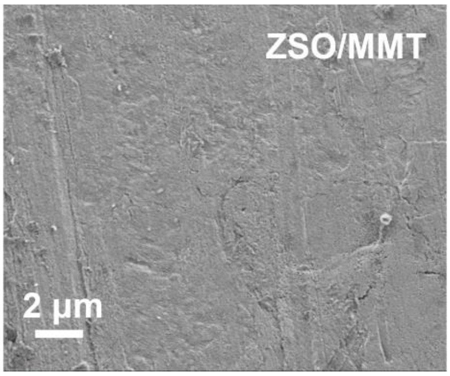

Figure S11. SEM image of Zn foil immersion in ZSO/MMT electrolyte for 7 days.

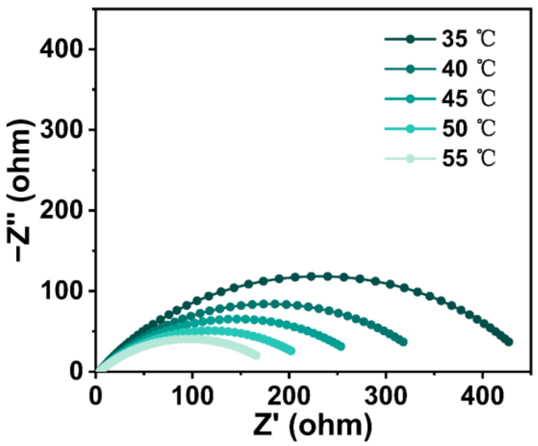

Figure S12. Nyquist plots of Zn||Zn symmetrical cells in ZSO/MMT electrolyte at different temperatures.

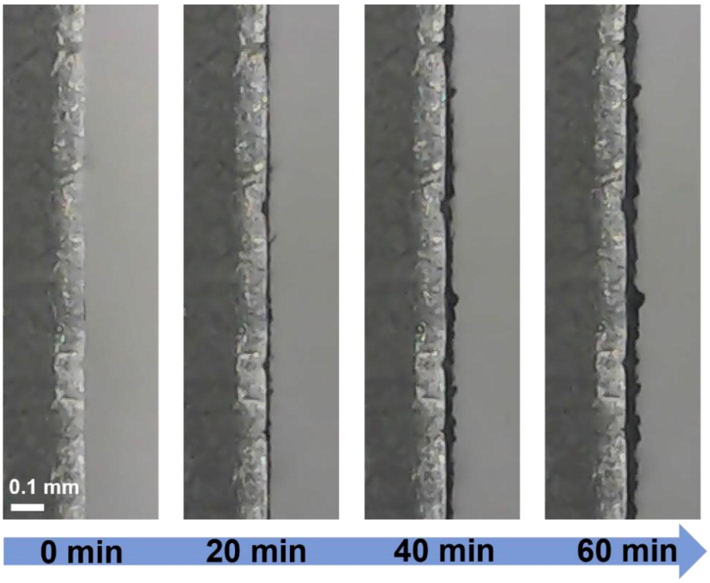

Figure S13. In situ operando optical microscope images of Zn plating in ZSO/MMT electrolyte.

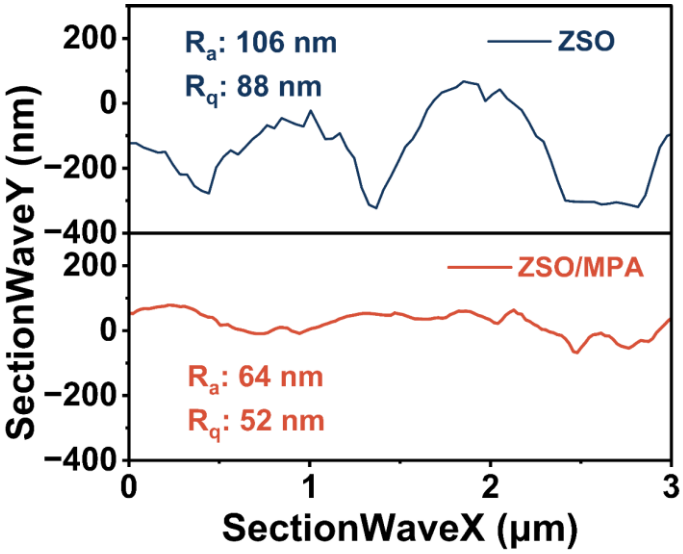

Figure S14. Surface roughness curve of the cycled Zn anode surfaces with different electrolytes.

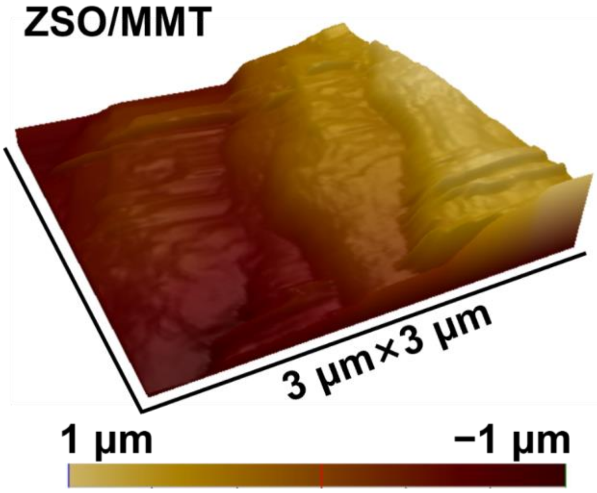

Figure S15. AFM images of the cycled Zn anode surface in ZSO/MMT electrolyte.

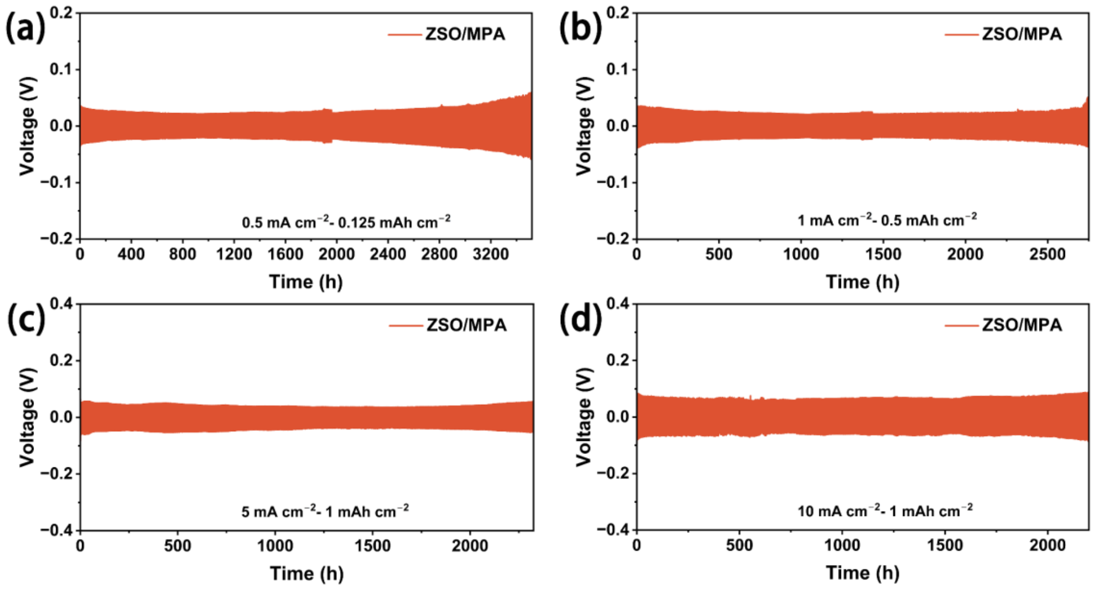

Figure S16. Long-term cycling performance of Zn||Zn symmetric cells in ZSO/MPA electrolyte at (a) 0.5 mA cm^{-2} and 0.125 mAh cm^{-2}, (b) 1 mA cm^{-2} and 0.5 mAh cm^{-2}, (c) 5 mA cm^{-2} and 1 mAh cm^{-2}, (d) 10 mA cm^{-2} and 1 mAh cm^{-2}.

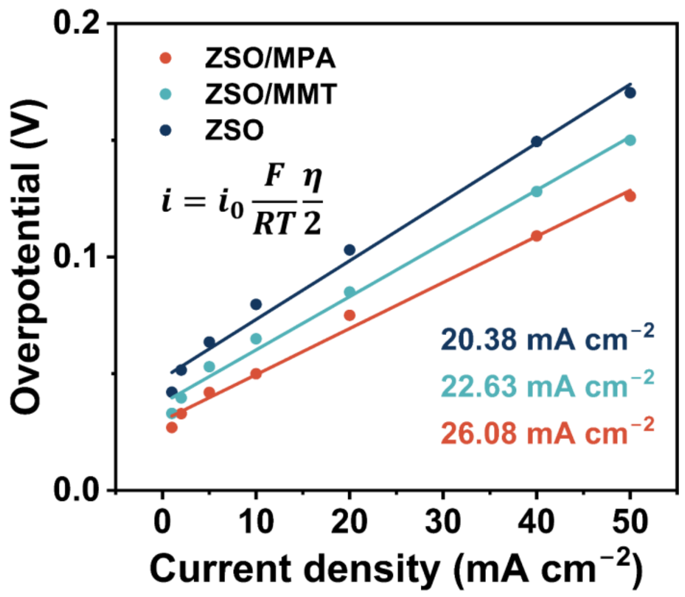

Figure S17. Exchange current densities of symmetric cells in different electrolytes.

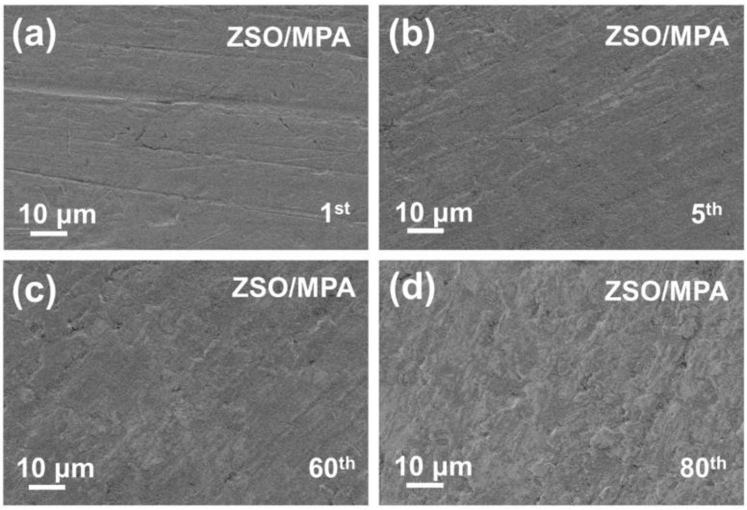

Figure S18. SEM image of Zn anode in ZSO/MPA electrolyte after different cycles.

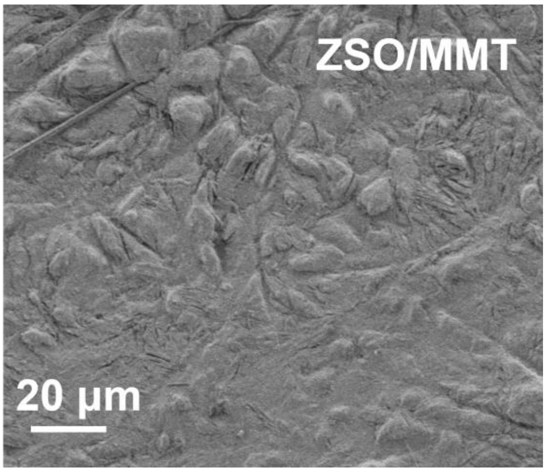

Figure S19. SEM image of Zn anode after 100 cycles in ZSO/MMT electrolyte.

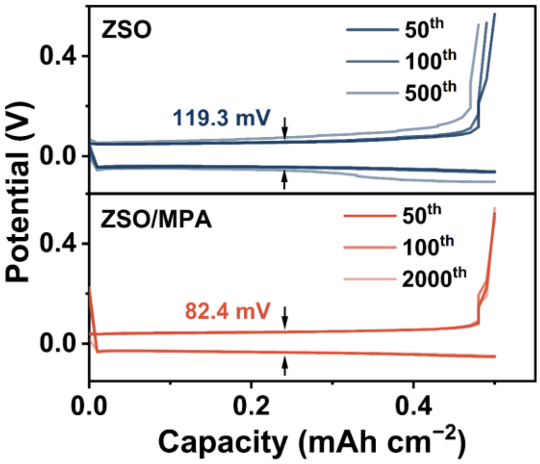

Figure S20. Voltage profiles of Zn||Cu cells in different electrolytes.

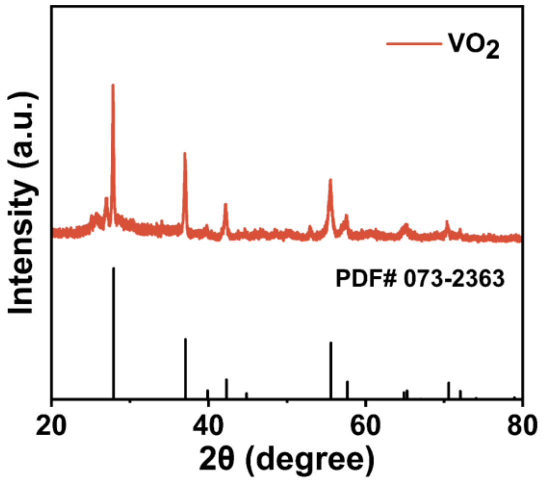

Figure S21. XRD pattern of synthesized VO_2 powders.

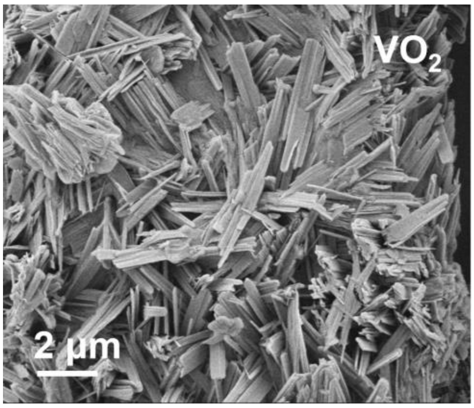

Figure S22. SEM image of VO_2.

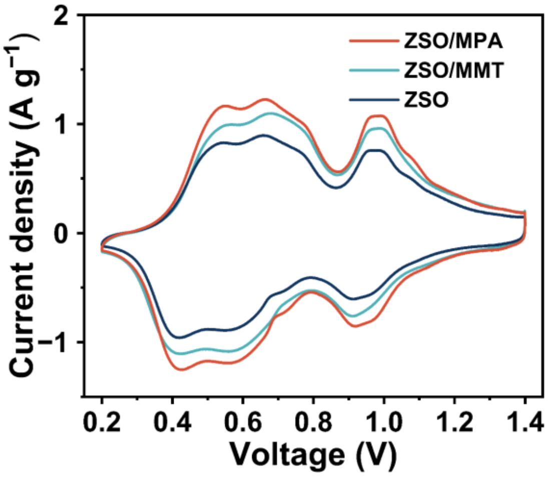

Figure S23. CV curves of full cells with different electrolytes.

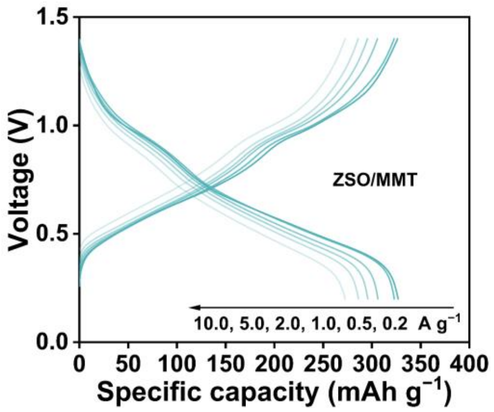

Figure S24. Charge-discharge curves of Zn full cell within ZSO/MMT electrolyte at various current densities.

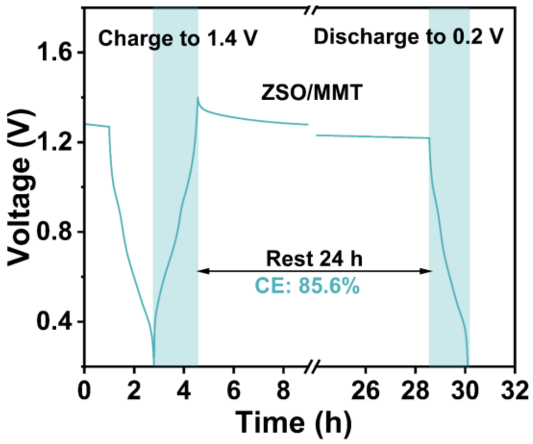

Figure S25. The self-discharge behavior of Zn||VO_2 full cell in ZSO/MMT electrolyte.

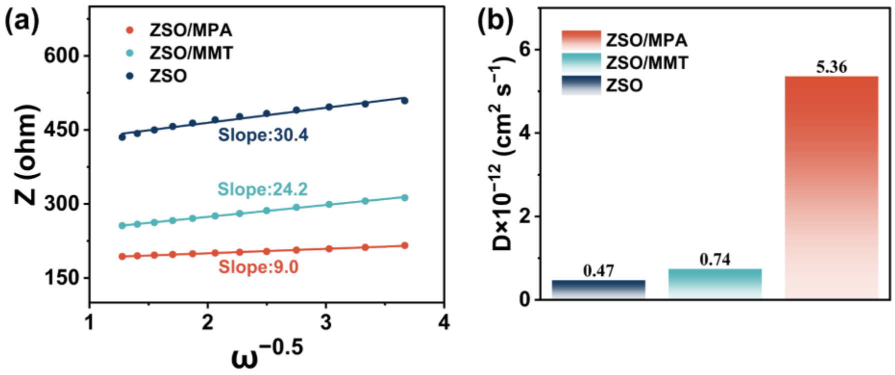

Figure S26. (a) Relationship of Z and $\omega^{-1/2}$ of full cells with different electrolytes in the low frequency region. (b) The diffusion coefficient of Zn^{2+} for the full cells within different electrolytes.

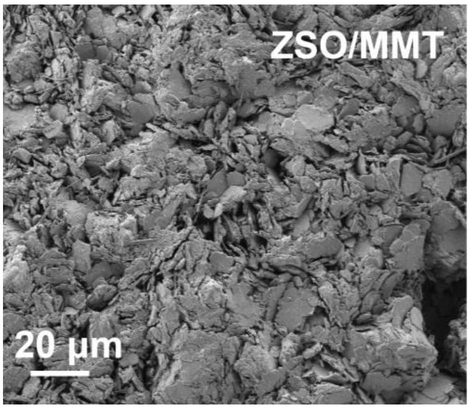

Figure S27. SEM image of Zn anode in ZSO/MMT electrolyte after 500 cycles at 5 A g^{-1}.

Table S1. Comparative analysis of cost/processing advantage.

Additives	Material	Cost (CNY/time)	Preparation
This work	Montmorillonite<br>Phytic acid	3.0	Stirring<br>Centrifugation<br>Suction filtration
α-ZrP	$Zr(HPO_4)_2 \cdot H_2O$<br>Ethanolamine	6.5	Stirring<br>Dialysis<br>Centrifugation
CDs	Na_4EDTA<br>NaCl	7.3	High-temperature sintering<br>Ultrasonication<br>Dialysis
C_3N_4	Melamine	6.0	High-temperature sintering<br>Ultrasonic cell disruptor<br>Centrifugation

Table S2. A summary of the values of R_{ct} for Zn symmetrical cells within different electrolytes at different temperatures.

Temperature (°C)	ZSO (Ω)	ZSO/MMT (Ω)	ZSO/MPA (Ω)
35	514.6	454.1	225.8
40	392.1	351.7	178.0
45	302.8	283.9	171.8
50	245.2	227.9	138.3
55	187.8	185.9	116.8

Table S3. Overpotentials of Zn symmetrical cells at different current densities within different electrolytes based on rate performance.

Current density (mA cm^{-2})	ZSO (mV)	ZSO/MMT (mV)	ZSO/MPA (mV)
1	42	33	27
2	52	40	33
5	64	53	42
10	80	65	50
20	103	85	75
40	150	128	109
50	170	150	126

Table S4. Comparison of electrochemical performances between symmetrical Zn||Zn cell in ZSO/MPA electrolyte and Zn anodes in other reported modified electrolytes.

Additives	Current density (mA cm^{-2})	Areal capacity (mAh cm^{-2})	Cumulative plating capacity (CPC) (Ah cm^{-2}) [2]	Lifespan (h)	Refs.
**MPA**	**0.5**	**0.125**	**0.875**	**3500**	**This work**
	**1**	**0.5**	**1.35**	**2700**	
	**5**	**1**	**5.75**	**2300**	
	**10**	**1**	**11**	**2200**	
BA	10	1	4	800	[3]
α-CD	10	1	0.8	160	[4]
NA	8	8	2.6	650	[5]
β-GP	3	1	2.7	1800	[6]
$MgSO_4$	1	0.25	0.3	600	[7]
AQS	0.5	0.5	0.625	2500	[8]
KPF_6	2	4	1.2	1200	[9]
CMC-Na	4	1	3.2	1600	[10]
TCFH	10	5	6.25	1250	[11]
$Zn(H_2PO_4)$	5	1	0.5	200	[12]
NaDFOB	5	1	1.25	500	[13]
ZHA	1	1	0.6	1200	[14]
TFEA	5	2	1.957	783	[15]
BBI	2	1	1.5	1500	[16]
DSF	5	5	3.38	1350	[17]
SPS	5	5	2.8	1120	[18]
His	5	5	2.5	1000	[19]
3TMS	4	4	1.672	836	[20]
POPSO-Na	2	1	1	1600	[21]
YTFPAA	0.5	0.25	0.525	2100	[22]
THF	1	1	1.4	2800	[23]

Supporting References

[1] F. Cui, J. Zhao, D. Zhang, Y. Fang, F. Hu, and K. Zhu, "VO_2(B) Nanobelts and Reduced Graphene Oxides Composites as Cathode Materials for Low-Cost Rechargeable Aqueous Zinc Ion Batteries," *Chemical Engineering Journal* 390, (2020): 124118. https://doi.org/10.1016/j.cej.2020.124118

[2] W. Fan, P. Li, J. Shi, et al., "Atomic Zincophilic Sites Regulating Microspace Electric Fields for Dendrite-Free Zinc Anode," *Advanced Materials* 36, no. 1 (2023): 2307219. https://doi.org/10.1002/adma.202307219

[3] Q. Guo, G. Teri, W. Mo, et al., "A Preferentially Adsorbed Layer on the Zn Surface Manipulating Ion Distribution for Stable Zn Metal Anodes," *Energy & Environmental Science* 17, no. 8 (2024): 2888-96. https://doi.org/10.1039/d4ee00986j

[4] K. Zhao, G. Fan, J. Liu, et al., "Boosting the Kinetics and Stability of Zn Anodes in Aqueous Electrolytes with Supramolecular Cyclodextrin Additives," *Journal of the American Chemical Society* 144, no. 25 (2022): 11129-37. https://doi.org/10.1021/jacs.2c00551

[5] H. Liang, J. Wu, J. Li, J. Wang, Z. Yang, and Y. Wu, "Achieving Dendrite-Free and by-Product-Free Aqueous Zn-Ion Battery Anode Via Nicotinic Acid Electrolyte Additive with Molecule-Ion Conversion Mechanism," *Small* 20, no. 38 (2024): 2402595. https://doi.org/10.1002/smll.202402595

[6] Y. Wang, T. Wang, P. Cui, et al., "Phosphated Electrolyte Enabling Dual Robust Electrode–Electrolyte Interfacial Reconstruction toward Capable Zn Metal Batteries," *Advanced Functional Materials* 35, no. 20 (2025): 2421363. https://doi.org/10.1002/adfm.202421363

[7] P. Wang, X. Xie, Z. Xing, et al., "Mechanistic Insights of Mg^{2+}-Electrolyte Additive for High-Energy and Long-Life Zinc-Ion Hybrid Capacitors," *Advanced Energy Materials* 11, no. 30 (2021): 2101158. https://doi.org/10.1002/aenm.202101158

[8] R. Sun, D. Han, C. Cui, et al., "A Self-Deoxidizing Electrolyte Additive Enables Highly Stable Aqueous Zinc Batteries," *Angewandte Chemie International Edition* 62, no. 28 (2023): e202303557. https://doi.org/10.1002/anie.202303557

[9] Y. Chu, S. Zhang, S. Wu, Z. Hu, G. Cui, and J. Luo, "In Situ Built Interphase with High Interface Energy and Fast Kinetics for High Performance Zn Metal Anodes," *Energy & Environmental Science* 14, no. 6 (2021): 3609-20. https://doi.org/10.1039/d1ee00308a

[10] S. Cui, X. Wang, W. Miao, et al., "Alleviating Zinc Dendrite Growth by Versatile Sodium Carboxymethyl Cellulose Electrolyte Additive to Boost Long-Life Aqueous Zn Ion Capacitors," *Energy Storage Materials* 68, (2024): 103356. https://doi.org/10.1016/j.ensm.2024.103356

[11] Z. Bao, Y. Wang, K. Zhang, et al., "Dual-Phase Interface Engineering Via Parallel Modulation Strategy for Highly Reversible Zn Metal Batteries," *Journal of Energy Chemistry* 101, (2025): 163-74. https://doi.org/10.1016/j.jechem.2024.09.053

[12] X. Zeng, J. Mao, J. Hao, et al., "Electrolyte Design for in Situ Construction of Highly Zn^{2+}-Conductive Solid Electrolyte Interphase to Enable High-Performance Aqueous Zn-Ion Batteries under Practical Conditions," *Advanced Materials* 33, no. 11 (2021): 2007416. https://doi.org/10.1002/adma.202007416

[13] Z. Wang, J. Diao, G. Henkelman, and C. B. Mullins, "Anion-Regulated Electric Double Layer and Progressive Nucleation Enable Uniform and Nanoscale Zn Deposition for Aqueous Zinc-Ion Batteries," *Advanced Functional Materials* 34, no. 24 (2024): 2314002. https://doi.org/10.1002/adfm.202314002

[14] J. Zheng, B. Zhang, X. Chen, et al., "Critical Solvation Structures Arrested Active Molecules for Reversible Zn Electrochemistry," Nano-Micro Letters 16, no. 1 (2024): 145. https://doi.org/10.1007/s40820-024-01361-0

[15] S. Yang, G. Wu, J. Zhang, et al., "A Stable High-Performance Zn-Ion Batteries Enabled by Highly Compatible Polar Co-Solvent," *Advanced Science* 11, no. 35 (2024): 2403513. https://doi.org/10.1002/advs.202403513

[16] J. Yu, F. Zhao, J. He, et al., "Regulating Zinc Hydroxide Sulfate (0 0 1) Preferential Growth and Zn (0 0 2) Deposition by Trace Dibenzenesulfonimide Additive toward Long Cycle Lifespan for Aqueous Zn-Ion Batteries," *Chemical Engineering Journal* 497, (2024): 154795. https://doi.org/10.1016/j.cej.2024.154795

[17] J. Li, S. Zhang, X. Yu, et al., "Enhancing Long-Term Cycling Stability in Aqueous Zinc-Ion Batteries Via Effective Control of Bulk Electrolyte with Biomass-Derived Multifunctional Additive," *Nano Energy* 141, (2025): 111049. https://doi.org/10.1016/j.nanoen.2025.111049

[18] T. Li, A. Naveed, J. Zheng, et al., "Engineering Aqueous Electrolytes with Vicinal S-Based Organic Additives for Highly Reversible Zinc-Ion Batteries," *Angewandte Chemie International Edition* 64, no. 21 (2025): e202424095. https://doi.org/10.1002/anie.202424095

[19]Q. Zong, B. Lv, Y. Yu, et al., "Close-Packed Growth and Buffer Action Enabling Stable and Reversible Zn Anode," *Nano Energy* 136, (2025): 110725 https://doi.org/10.1016/j.nanoen.2025.110725

[20] Q. Xiao, S. He, P. Liu, et al., "Multifunctional Silanol-Based Film-Forming Additive for Stable Zn Anode," *Advanced Functional Materials* 35, no. 12 (2024): 2417708. https://doi.org/10.1002/adfm.202417708

[21] H. Peng, D. Wang, X. Wang, et al., "Coupling Solvation Structure Regulation and Interface Engineering Via Reverse Micelle Strategy toward Highly Stable Zn Metal Anode," *Advanced Functional Materials* 35, no. 12 (2024): 2417695. https://doi.org/10.1002/adfm.202417695

[22] L. Li, C. Chen, P. Meng, Y. Zhang, and Q. Liang, "A Water-Insoluble Yttrium-Based Complex as Dual-Ionic Electrolyte Additive for Stable Aqueous Zinc Metal Batteries," *Advanced Functional Materials* 34, no. 42 (2024): 2406965. https://doi.org/10.1002/adfm.202406965

[23] S. You, Q. Deng, Z. Wang, et al., "Achieving Highly Stable Zn Metal Anodes at Low Temperature Via Regulating Electrolyte Solvation Structure," *Advanced Materials* 36, no. 26 (2024): 2402245. https://doi.org/10.1002/adma.202402245
